# Water‐Soluble Polymeric Carbon Nitride Colloidal Nanoparticles for Highly Selective Quasi‐Homogeneous Photocatalysis†

**DOI:** 10.1002/anie.201913331

**Published:** 2019-11-26

**Authors:** Igor Krivtsov, Dariusz Mitoraj, Christiane Adler, Marina Ilkaeva, Mariana Sardo, Luís Mafra, Christof Neumann, Andrey Turchanin, Chunyu Li, Benjamin Dietzek, Robert Leiter, Johannes Biskupek, Ute Kaiser, Changbin Im, Björn Kirchhoff, Timo Jacob, Radim Beranek

**Affiliations:** ^1^ Department of Organic and Inorganic Chemistry University of Oviedo-CINN 33006 Oviedo Spain; ^2^ Institute of Electrochemistry Ulm University Albert-Einstein-Allee 47 89081 Ulm Germany; ^3^ CICECO—Aveiro Institute of Materials Department of Chemistry University of Aveiro Campus Universitário de Santiago 3810-193 Aveiro Portugal; ^4^ Institute of Physical Chemistry and Abbe Center of Photonics Friedrich Schiller University Jena Lessingstrasse 10 07743 Jena Germany; ^5^ Center for Energy and Environmental Chemistry Jena (CEEC Jena) Philosophenweg 7a 07743 Jena Germany; ^6^ Department Functional Interfaces Leibniz Institute of Photonic Technology (IPHT) Albert-Einstein-Strasse 9 07745 Jena Germany; ^7^ Electron Microscopy of Materials Science, Central Facility for Electron Microscopy Ulm University Albert-Einstein-Allee 11 89081 Ulm Germany; ^8^ Science Institute University of Iceland Dunhaga 5 107 Reykjavík Iceland; ^9^ Helmholtz-Institute-Ulm (HIU) Helmholtzstrasse 11 89081 Ulm Germany; ^10^ Karlsruhe Institute of Technology (KIT) P.O. Box 3640 76021 Karlsruhe Germany

**Keywords:** carbon nitride, chemoselectivity, nanoparticles, photocatalysis, quasi-homogeneous catalysis

## Abstract

Heptazine‐based polymeric carbon nitrides (PCN) are promising photocatalysts for light‐driven redox transformations. However, their activity is hampered by low surface area resulting in low concentration of accessible active sites. Herein, we report a bottom‐up preparation of PCN nanoparticles with a narrow size distribution (ca. 10±3 nm), which are fully soluble in water showing no gelation or precipitation over several months. They allow photocatalysis to be carried out under quasi‐homogeneous conditions. The superior performance of water‐soluble PCN, compared to conventional solid PCN, is shown in photocatalytic H_2_O_2_ production via reduction of oxygen accompanied by highly selective photooxidation of 4‐methoxybenzyl alcohol and benzyl alcohol or lignocellulose‐derived feedstock (ethanol, glycerol, glucose). The dissolved photocatalyst can be easily recovered and re‐dissolved by simple modulation of the ionic strength of the medium, without any loss of activity and selectivity.

## Graphical abstract

**Quasi‐modus**: Water‐soluble colloidal nanoparticles of polymeric carbon nitride enable quasi‐homogeneous photocatalysis with unprecedented performance. The dissolved photocatalyst can be easily recovered and re‐dissolved by simple modulation of the ionic strength of the medium, retaining its excellent activity and selectivity.

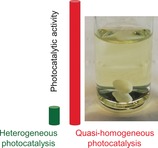

## Introduction

Since the discovery of the photocatalytic activity of polymeric carbon nitride (PCN),^1^ numerous top‐down^2^ and bottom‐up^3^ synthetic strategies aiming to disrupt the strong hydrogen bonding and van der Waals stacking in bulk‐PCN have been reported. These efforts aim at improving the dispersibility of PCN in the reaction medium and to enhance the photocatalytic performance by increasing the number of accessible surface active sites. While top‐down methods (e.g., sonication, heat and/or acid treatment) providing low‐molecular weight materials were at least partially effective for achieving this goal, they unavoidably led to reduced yields of the catalyst due to losses occurring during exfoliation^4^ or etching^5^ and could not achieve truly homogeneous nanoparticles solutions. Another approach is based on breaking of H‐bonds in bulk‐PCN by dissolving it in appropriate solvents such as concentrated sulfuric acid^6^ or sulfonic acid derivatives,^7^ which destroys the polymeric structure leading to the formation of discrete PCN species (less than ca. 1 nm size) and limits the application of the dissolved photocatalyst to aggressive media. Highly alkaline conditions were also applied to obtain water‐soluble PCN, however, no photocatalytic activity of the derived gels was reported.^8^ Recent efforts regarding bottom‐up synthesis of water‐soluble or highly‐dispersed PCN include templating,^9^ high‐temperature synthesis in salt melts,^10^ electrochemical poly(triazine imide) preparation,^11^ sol‐gel chemistry using self‐induced gelation, occurring within several days, of poly(heptazine imide) hydrosols prepared by the KSCN‐assisted condensation of melamine,^12^ and formation of water‐soluble PCN “quantum dots”, which were highly luminescent, but found to be photocatalytically inactive^13^ or their photocatalytic activity was not reported.2b, 3b, 14

Herein, we report, for the first time, a facile and high‐yield preparation of PCN nanoparticles with a narrow size distribution (ca. 10±3 nm), which are fully soluble in water showing no gelation or precipitation over several months, and allow carrying out highly efficient photocatalysis under quasi‐homogeneous conditions. The enhanced performance of this material, as compared to the conventional solid PCN, is demonstrated in dual‐product photocatalysis involving highly selective (up to 100 %) photooxidation of one of the lignin model compounds 4‐methoxybenzyl alcohol (4MBA)^15^ to 4‐methoxybenzaldehyde (4MBAL), concurrent with highly selective (≥70 %) reduction of O_2_ to H_2_O_2_, another highly valuable chemical feedstock with a market price higher than, for example, methanol. In addition, the superior photoactivity of water‐soluble PCN is confirmed by enhanced H_2_O_2_ production rates using also benzyl alcohol and lignocellulose‐derived feedstock as alternative reducing agents (ethanol, glycerol, glucose). Finally, we provide a very simple protocol for recovery and re‐dissolution of the water‐soluble photocatalyst, which enables easy handling and excellent recyclability of our photocatalyst under quasi‐homogeneous operation conditions without any apparent loss of activity and selectivity.

## Results and Discussion

### Synthesis and Characterization

**Figure 1:**
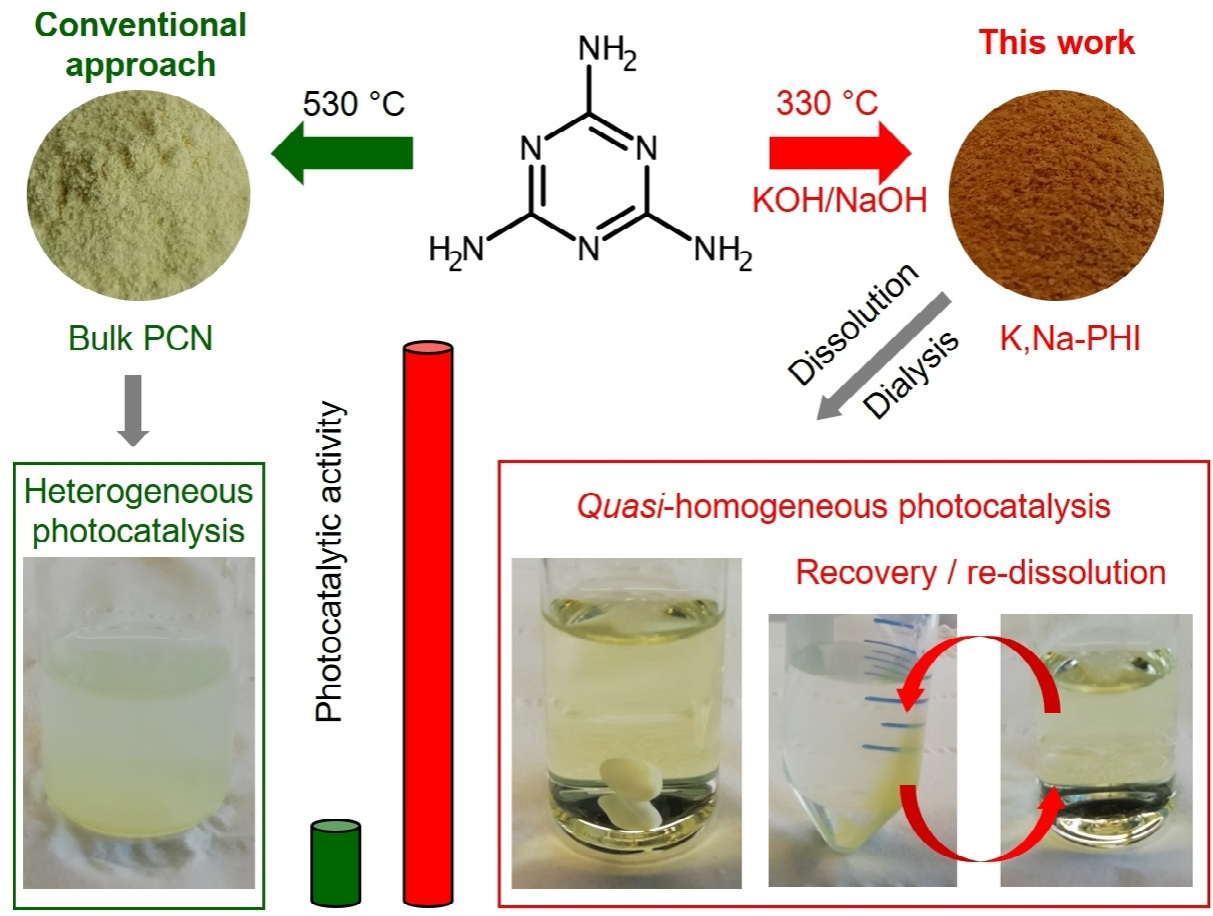
Alkali poly(heptazine imide) is synthesized at comparatively low temperature (330 °C) using a KOH/NaOH melt; after dissolution and dialysis the resulting material is a water‐soluble photocatalyst, more active than the bulk PCN, and easily recovered (by increasing the ionic strength via addition of NaCl) and dissolved again, thus enabling recyclability without any loss of activity and selectivity.

Figure 1 shows a schematic representation of the synthesis procedure resulting in a highly active water‐soluble and recoverable PCN photocatalyst. Instead of using conventional metal halide‐based solvents applied for the preparation of poly(triazine imides) and poly(heptazine imides),^16^ or alkali‐metal hydroxides at high temperature,^17^ the use of a KOH/NaOH melt allowed us to reduce the temperature of melamine condensation to PCN structures from the usual range of 500–600 °C, or 450 °C reported by Wang et al. for the case of KSCN solvent,^12^ down to 330 °C.

**Figure 2:**
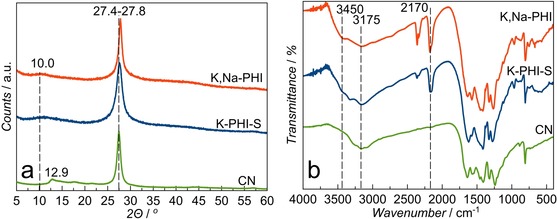
XRD patterns (a) and FTIR spectra (b) of the materials measured after dialysis and drying.

The use of KOH at loadings as low as 12.5 mmol (per 1.5 g ca. 12 mmol melamine) in the synthesis yields mostly insoluble potassium poly(heptazine imide) (K‐PHI‐S) (see Supporting Information, Table S1), while higher loadings (above 15 mmol) result in the formation of a pale‐yellow potassium poly(heptazine imide) solution (K‐PHI) with very low yield (<9 %). The use of NaOH leads to a water‐soluble sodium poly(heptazine imide) (Na‐PHI) even at low NaOH quantities (5 mmol), most likely due to its lower melting point, while higher concentrations in the melt induce the degradation of melamine and, consequently, a drastic decrease of the poly(heptazine imide) yield (<4 %; Table S1). The yields of the corresponding dialysed and dried material were highest for melamine condensation in a 10 mmol:5 mmol KOH/NaOH melt (yields up to 42 %). The resulting mixed potassium/sodium poly(heptazine imide) (K,Na‐PHI) material is water‐soluble with a clearly observable Tyndall effect (Figure S1). Thermal analysis of the precursor mixture (melamine + KOH/NaOH) shows two main stages of mass loss at 150 and 280 °C assigned to H_2_O evolution and to melamine deammonification, respectively (Figure S2). The presence of hydroxides in the melt catalyses the condensation of melamine, thus reducing the synthesis temperature of poly(heptazine imide) to 330 °C. This is, to our knowledge, the lowest temperature reported for the solid‐state synthesis of heptazine‐based PCN materials. The obtained solid (K‐PHI‐S) and dried solutions of the poly(heptazine imide) after dialysis (K,Na‐PHI) show XRD patterns typical for conventional bulk melamine‐derived PCN (melon, here designated as CN sample) and low‐crystalline poly(heptazine imide) materials with an interlayer spacing of about 3.0 Å and a (100) reflection at 8.9 Å resulting from alkali metal cations incorporated into the poly(heptazine imide) structure^18^ (Figure 2 a, Figures S3, S4). The structure of the prepared solid K‐PHI‐S and dried K,Na‐PHI samples is also confirmed by FTIR spectra showing a typical fingerprint of PCN materials in the range of 1200 to 1700 cm^−1^, corresponding mostly to υ(C‐NH‐C) and υ(C=N) stretching vibrations, and a peak at 800 cm^−1^ attributed to the triazine ring breathing mode.^18^ The peak at 3450 cm^−1^, visible only for K,Na‐PHI and K‐PHI‐S (Figure 2 b), may be assigned to the stretching vibrations of OH‐groups incorporated into the poly(heptazine imide) structure as a result of the alkali melt treatment. Additionally, in the range 2150–2175 cm^−1^, υ(C≡N) vibrations are clearly present in the spectra of the samples prepared in hydroxide melts (Figure 2 b). The same peak assignments can be made regarding the FTIR spectra of K‐PHI and Na‐PHI (Figure S5).

From all the synthesised series the samples K‐PHI‐S and K,Na‐PHI were chosen for the detailed investigation as being the representatives of insoluble and soluble poly(heptazine imides; Table S1). These materials possess a high thermal stability typical for other PCNs, starting to decompose only at temperatures of about 600 °C (Figure S6). CHN elemental analysis of the K,Na‐PHI and K‐PHI‐S samples shows C:N ratios of 0.67 and 0.72, respectively, which are typical for poly(heptazine imide) materials. The reduced concentration of H and the increased C:N ratio observed for K,Na‐PHI might indicate a partial loss of NH_2_‐containing species due to polymer hydroxylation and/or lower fragmentation of the polyheptazine network (Table S2). The EDX elemental analysis reveals that the content of alkali metals and oxygen is doubled in K,Na‐PHI with respect to K‐PHI‐S (Table S2). This might be attributed not only to the higher number of surface oxygen‐containing functional groups, but also to increased amounts of chemisorbed water and CO_2_, whose presence might be responsible for the formation of alkali‐metal hydroxides or carbonates melting at high temperature (Figure S6).

**Figure 3:**
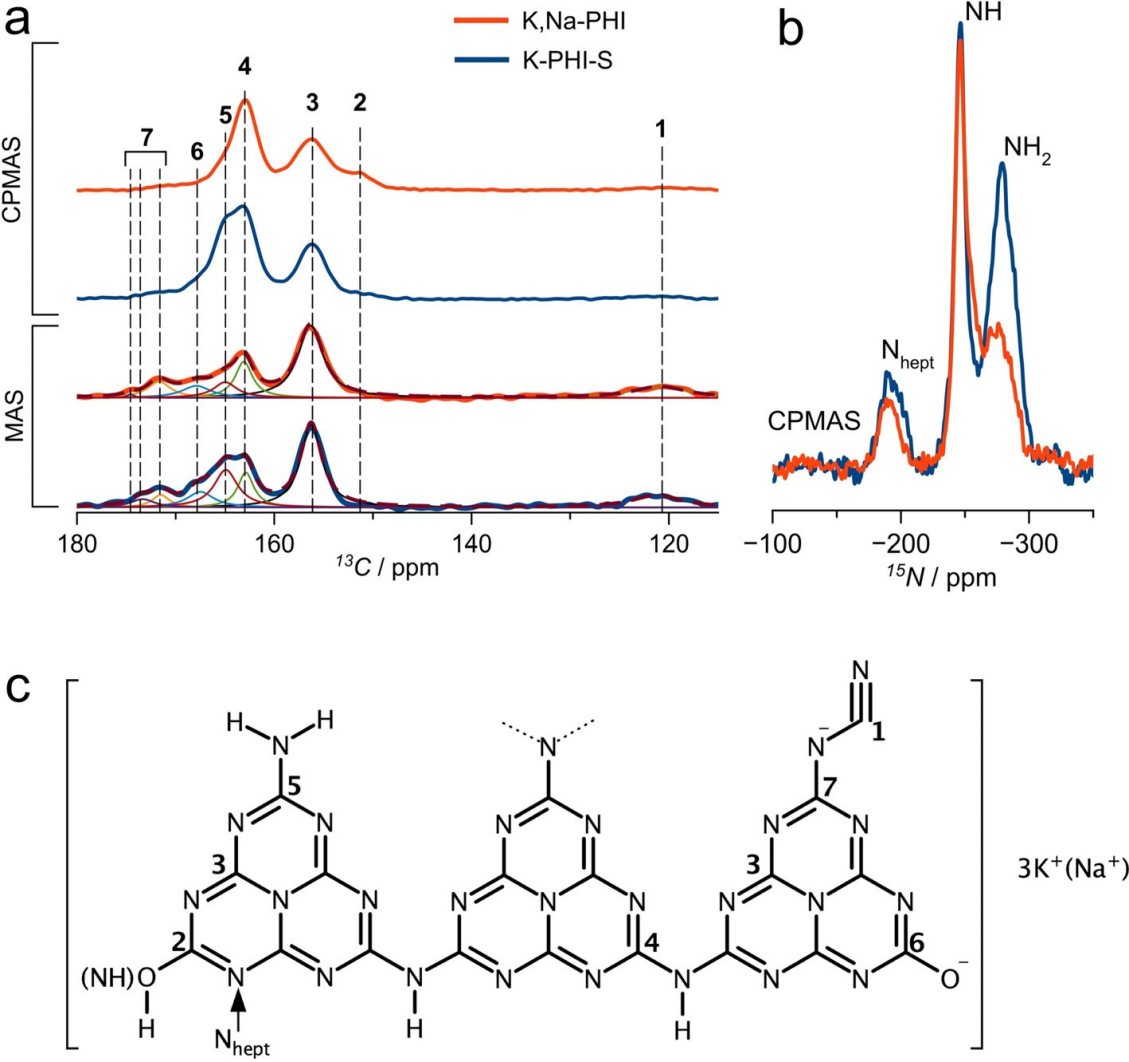
a) ^13^C direct excitation MAS and ^1^H‐^13^C CPMAS b) ^1^H‐^15^N CPMAS NMR spectra of K‐PHI‐S and K,Na‐PHI and c) schematic representation of the functionalized PCN and atom numbering scheme. ^13^C MAS spectra (a) have been deconvoluted showing the different components assigned to the carbon atoms depicted in (c).

To obtain further insight into the chemical structure of the materials, solid‐state NMR analysis was carried out. ^13^C NMR direct excitation of the K,Na‐PHI sample shows a series of resonances at 122, 156, 163, 165, 168, and 169–174 ppm, which are assigned to the carbon atoms in C≡N (**1**), N−C=N (**3**), C‐N_2_(NH) (**4**), C‐N_2_(NH_2_) (**5**), C‐O^−^ (**6**) and C‐N^−^ groups (**7**), likely found in different environments (Figures 3 a, c, Figure S7).^16^, ^19^ The presence of CO_3_^2−^ species cannot be excluded as its chemical shift coincides with resonances **6** and **7**. The presence of non‐protonated N−C=N (**3**) groups suggests the poly(heptazine) nature of the material, although a possible admixture of poly(triazine) units cannot be ruled out completely. In ^1^H‐^13^C cross‐polarization (CP) magic‐angle spinning (MAS) NMR spectra of K‐PHI‐S and K,Na‐PHI the resonances ascribed to carbons **4** and **5** are significantly increased with respect to the other resonances (CPMAS and MAS, Figure 3 a), while this is not the case for resonances **1**, **3**, **6**, and **7**. A new resonance emerges at 151 ppm (**2**) in the ^1^H‐^13^C CPMAS spectrum of the K,Na‐PHI sample, while it is less pronounced for the K‐PHI‐S sample (Figures 3 a,c, Figure S7). This resonance is tentatively attributed either to the C‐OH group of the aromatic poly(heptazine imide) structure or to the deprotonated analogous, that is, C‐O^−^ moieties H‐bonded to NH_*x*_ resulting from drying and polymerization. A similar observation was made for crystalline potassium cyamelurates and cyameluric acid.^20^ A distinguishable shoulder, at 165 ppm (**5**), is observed at the left side of the ^13^C resonance **4** (ca. 163 ppm) for K‐PHI‐S (Figure 3 a), which corresponds to N_2_C‐(NH_2_) and N_2_C‐(NH) species, respectively. Notably, the resonance at 165 ppm (**5**), observed in K‐PHI‐S, is drastically reduced for K,Na‐PHI (Figures 3 a,c, Figure S7). Resonance **6** at about 168 ppm, observed from the ^13^C MAS spectrum of K,Na‐PHI is not enhanced on the ^1^H‐^13^C CPMAS spectrum, suggesting the lack of protons in the vicinity of this functional group. This supports the assignment of **6** to C‐O^−^ stabilized by K^+^ or Na^+^, such as in the [C_6_N_7_O_3_]^3−^ groups of K_3_[C_6_N_7_O_3_]×H_2_O.^21^ The ^1^H‐^15^N CPMAS spectra of K,Na‐PHI and K‐PHI‐S show three resonances at −275, −246, and −191 ppm assigned to NH_2_, NH and heptazine secondary amines (N_hept_), respectively. Although CPMAS is not quantitative, the NH_2_/NH intensity ratio of the K,Na‐PHI sample is much smaller than that of K‐PHI‐S; we hypothesize that NH_2_ groups are consumed due to reaction with KOH/NaOH (Figure 3 b,c). Also, a shoulder at −257 ppm, attributed to ‐N=C‐OH(NH) can be observed in the spectrum of the K,Na‐PHI sample (Figure S7). A quantitative estimation based on the ^13^C direct excitation MAS spectra (Supporting Information, NMR study, Table S3, Figure S7) suggests that the formation of heptazine species (Figure S8) is favoured for the case of the K,Na‐PHI sample, resulting in an increased number of N−C=N (**3**) carbons in K,Na‐PHI as compared to K‐PHI‐S (Table S3, Figure S7). The lower concentration of terminal ‐NH_2_ moieties in K,Na‐PHI will likely lead to an overall decrease of the number of hydrogen bonds in the polymer network, thus exerting a direct influence on aggregation and solubility.

The X‐ray photoelectron spectroscopy (XPS) data corroborate the presence of C‐O^−^ species in both analysed samples (Figure S9, Table S4) and indicate the redistribution of N‐containing groups for the material prepared using KOH/NaOH mixture (Figure S9 e,f, Table S4). Although C1s XP spectra confirm the presence of N−C=N bonds, they do not allow unambiguous assignment of the peak at 286.2 eV that may be attributed to C‐NH_*x*_ and C‐O^−^ groups (Figure S9, Table S4). The N1s spectra reveal a different distribution of the N‐containing species in K‐PHI‐S and K,Na‐PHI. Based on integrated peak intensities, the increased contribution of the peak (C−N=C plus C≡N (N1)) with respect to those attributed to N‐C_3_ plus triazine C‐NH_*x*_ (N2) and heptazine C‐NH_*x*_ (N3) suggests a higher relative content of N belonging to the PCN aromatic structure in the K,Na‐PHI material. This indicates a higher degree of condensation (Figure S9, Table S4). The O1s spectrum also exhibits an additional band at 531.0 eV in K,Na‐PHI which can be tentatively assigned to CO_2_ chemisorbed by the alkali metal ions present in higher quantities than in the K‐PHI‐S sample or to the hydroxylated carbon nitride species.

Summarizing, we hypothesize that the insoluble K‐PHI‐S material prepared in KOH melt has a poly(heptazine imide) structure bearing NH, ‐NH_2_ groups, uncondensed NH_*x*_‐containing triazine species and oxygenated surface moieties in the form of cyamelurate fragments. The water‐soluble K,Na‐PHI prepared in KOH/NaOH mixture is also composed of a poly(heptazine imide) network. However, it is suggested to have a higher number of heptazine nitrogen atoms owing to a higher degree of triazine‐to‐heptazine condensation and a much smaller number of terminal amines resulting in weaker H‐bonding between the poly(heptazine) layers. This, in combination with the presence of terminal cyamelurate functional groups in the material facilitating the formation of a solvent structure around the K,Na‐PHI particles, rationalizes its high solubility in water. The dissolved K,Na‐PHI has a zeta potential of −31 mV at pH of 7.5; this is a typical value for PCN‐derived materials^22^ which are prone to agglomeration in suspensions due to strong H‐bonding between the terminal NH_*x*_ species and the N atoms of the heptazine units. This means that electrostatic repulsion alone cannot be entirely responsible for the excellent dispersion of K,Na‐PHI in water. We speculate that in K,Na‐PHI, the cyamelurate C‐O^−^ groups are stabilized by H‐bonds mainly with water molecules from the well‐ordered hydration shell of the Na^+^ ion. At low concentrations, Na^+^ ions will therefore promote formation of a hydration layer at the PHI surface which improves solubility. With increasing Na^+^ concentration the hydration layer will gradually be stripped causing coalescence due to positive van der Waals forces (see Figure S10). Notably, we have not found any appreciable solubility of K,Na‐PHI in most of the common polar or non‐polar organic solvents. This corroborates our assumption that the cyamelurate‐functionalities of K,Na‐PHI specifically interact with water creating a water solvation shell, which hinders the formation of H‐bonds with other dissolved species and increases the electrostatic repulsion between nanoparticles due to the negative surface charge.

DFT calculations using an implicit solvation approach on a periodic PHI model are in agreement with this hypothesis (see Supporting Information, Theoretical study of K,Na‐PHI). By comparing the stability of the model system (Figure S11) in water, acetonitrile, or methanol environments, we find that water will lead to a higher stabilization than acetonitrile, or methanol (Table S5). Further, we find that stabilization increases with the number of oxygen‐containing (exchanged for –NH_2_) moieties.

**Figure 4:**
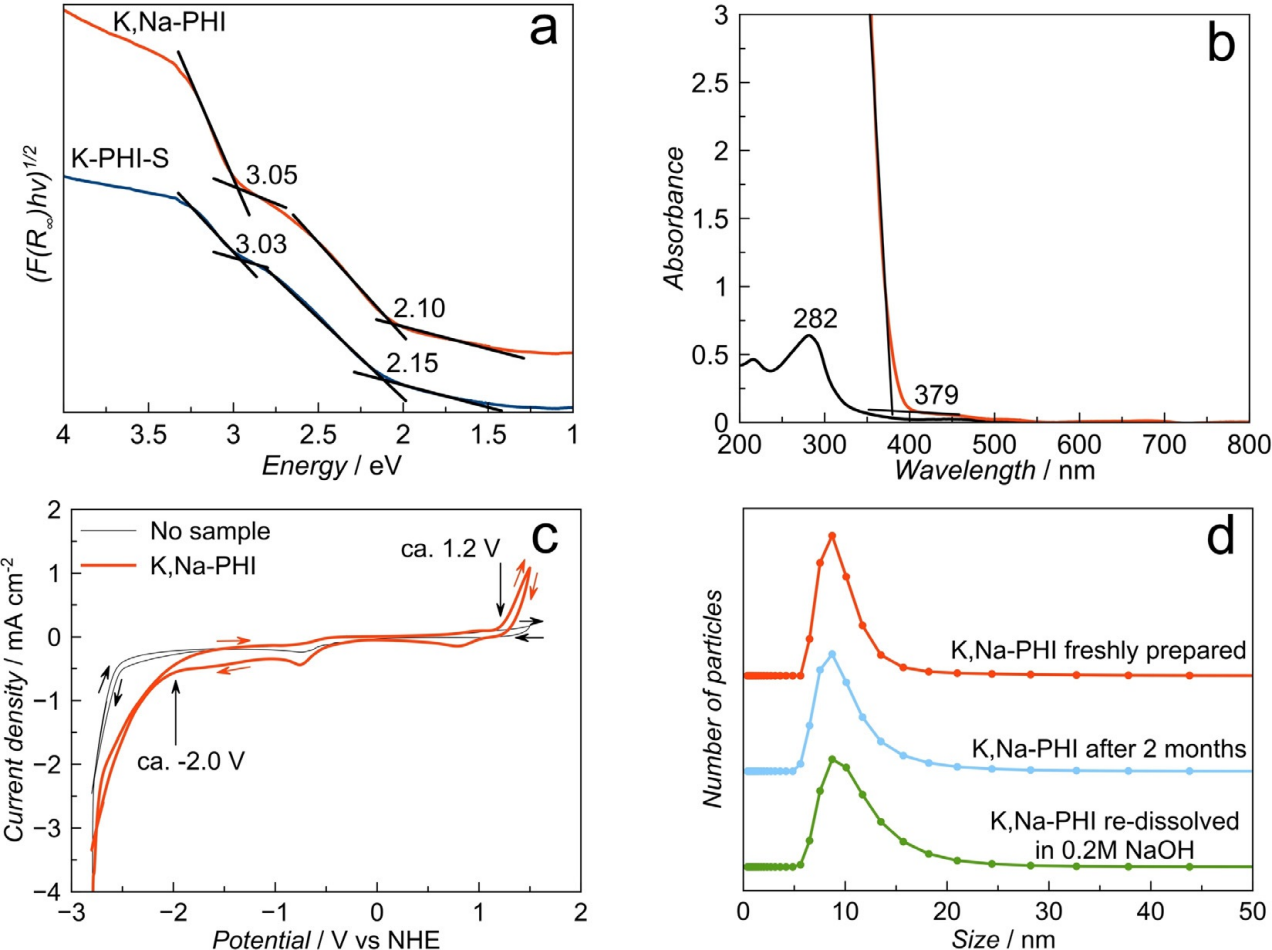
a) Diffuse reflectance UV/Vis spectra of the solid K‐PHI‐S and dried K,Na‐PHI samples transformed using the Tauc formalism for the determination of absorption edges. b) UV/Vis electronic absorption spectra of K,Na‐PHI dissolved in water at concentrations of 0.005 and 0.75 g L^−1^. c) Cyclic voltammetry (50 mV s^−1^, 0.1 m TBAPF_6_ in acetonitrile) of the K,Na‐PHI sample deposited on a glassy carbon electrode. d) Particle size distribution of the K,Na‐PHI samples measured by DLS.

The analysis of electronic absorption properties of the dried poly(heptazine imide) samples using diffuse reflectance spectroscopy shows the presence of two optical absorption edges at 2.1–2.2 and 3.0 eV (Figure 4 a). In contrast, the electronic absorption UV/Vis spectra taken from dissolved K,Na‐PHI in water do not exhibit any significant light absorption in the visible range (Figure 4 b). However, it is clear that a maximum absorption edge appears in the UV range (285 nm) under dilute conditions, while in concentrated solution the absorption edge shifts to 379 nm corresponding to about 3.3 eV (Figure 4 b). The absorption at 285 nm can be attributed to the cyamelurate species present in K,Na‐PHI.20a The slightly lower‐energy absorption edge value of 3.0 eV measured in the solid‐state as well as the appearance of the additional absorption edge at 2.1 eV might be attributed to the stacking or coalescence of the poly(heptazine imide) units, contrary to what is observed for the well‐separated nanoparticles in a colloidal solution. The cyclic voltammetry study of the K,Na‐PHI sample is in agreement with the energy gap determined by the optical method, showing the HOMO and LUMO positions at 1.2 V and −2.0 V versus normal hydrogen electrode (NHE), respectively (Figure 4 c). The water‐soluble K,Na‐PHI nanoparticles prepared in this work show a relatively narrow size distribution (ca. 6–20 nm) as determined by dynamic light scattering (DLS; Figure 4 d). The mean particle size of the sample is around 10±3 nm (Figure 4 d). The alkali poly(heptazine imide) materials prepared using only NaOH or only KOH (Na‐PHI or K‐PHI) demonstrate slightly broader size distribution (Figure S12). The Na,K‐PHI particles are stable in water‐based media for several months without suffering agglomeration. Notably, the dried material can also be re‐dissolved in 0.2 m NaOH exhibiting a nearly identical particle size distribution, which confirms that the water‐soluble PCN nanoparticles retain their morphological and structural integrity (Figure 4 d).

The coalescence of dissolved particles after drying results in almost featureless SEM images, not showing any definite morphology (Figure S13). Although we were unable to obtain high‐resolution TEM images due to significant beam damage to the samples, fibre‐like morphology of the sample prepared from a dilute solution of K,Na‐PHI dried on the microscope grid is noted (Figure S14). The thickness of the fibres is estimated to be about 10 nm, which is in agreement with the data from dynamic light scattering (Figure 4 d). Note, however, that it cannot be ruled out that the observed morphology results from coalescence of the particles during the drying on the grid. We speculate that fibre net observed in TEM image is a result of the anisotropy of the original PHI particles causing directional agglomeration.

### Photocatalytic studies

**Figure 5:**
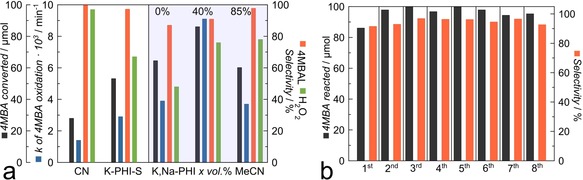
a) Photocatalytic oxidation of 4MBA with simultaneous H_2_O_2_ formation in H_2_O (20 mL, 4MBA 0.1 mmol, pH 6.5–7.0, LED 365 nm, 4 h) and in H_2_O/MeCN mixture in the presence of K,Na‐PHI (20 mL, *x* vol % of MeCN, 4MBA 0.1 mmol, LED 365 nm, 4 h) b) recyclability test of the K,Na‐PHI samples for 4MBA oxidation (20 mL, H_2_O/MeCN(40 vol %), 4MBA 0.1 mmol, pH 6.5–7.0, LED 365 nm, 4 h, 0.125 g of NaCl is added for the photocatalyst recovery).

Pristine and modified PCNs are well‐known photocatalysts for partial photooxidation of organic molecules,^23^ and methoxy‐substituted aromatic alcohols in particular,^24^ which opens the route for synthesis of high‐value chemicals from low‐value feedstock using only sunlight or cost‐effective light sources (e.g., LED arrays) and easily available oxidizing agents such as aerial oxygen. The water‐soluble colloidal PCN nanoparticles prepared in this work allow, for the first time, photocatalysis to be performed with PCN under quasi‐homogeneous conditions. The optimization of the photocatalyst concentration in the reaction mixture was carried out for the solid K‐PHI‐S and the water soluble K,Na‐PHI samples, resulting in optimal loadings of 0.5 g L^−1^ and 0.75 g L^−1^, respectively (Figures S15, S16). The photocatalytic performance of other water‐soluble samples (K‐PHI and Na‐PHI) was lower or equal to that of K,Na‐PHI (Figure S17), henceforth we focus on the photocatalytic properties of K,Na‐PHI, which can be obtained in much higher synthetic yields. Initial tests for selective photooxidation of 4MBA to 4MBAL in water have demonstrated increased activity of K‐PHI‐S and K,Na‐PHI with respect to the conventional PCN (CN sample) by factors of 2.1 and 2.8 under heterogeneous and quasi‐homogeneous conditions, respectively (Figure 5 a, Figure S18, Table S6). Notably, formation of the aldehyde is accompanied by two‐electron reduction of O_2_, producing hydrogen peroxide,^25^ another highly valuable compound. The control experiments in the dark, with the K,Na‐PHI and CN photocatalysts as well as 4MBA photolysis in the absence of any photocatalysts, showed that 4MBA oxidation was negligible under such conditions, and no formation of H_2_O_2_ was observed (see Supporting information). Additional control experiments under the flow of argon (to avoid the presence of oxygen) showed, as expected, that the H_2_O_2_ production was negligible (Figure S19). The 4MBAL production dropped significantly (by >60 %) and was clearly accompanied by reduction of the photocatalyst as its colour turned from yellow to brown, whereby this colour change disappeared after the exposure to air. This is an indication of photogenerated electrons being stored in the form of K,Na‐PHI radicals, which was reported earlier for this type of materials.^26^

Using a purely aqueous solvent, the selectivity towards both target molecules after 4 h of irradiation is lower for the most active K,Na‐PHI sample as compared to the benchmark CN photocatalyst (Figure 5 a, Figure S18, Table S6). Addition of acetonitrile (MeCN), whereby the quasi‐homogeneous reaction conditions are retained below 50 vol% of MeCN (Figures S20, S21), was found to significantly increase both activity and selectivity. We hypothesise that addition of MeCN accelerates desorption of the less polar aldehyde from the K,Na‐PHI surface (Figure 5 a, Figure S18, Table S6). The heterogenization of the reaction medium occurring at concentrations of MeCN >50 vol% (Figures S20, S21) is detrimental for the photocatalytic activity (Figure 5 a, Figure S18, Table S6), which confirms the beneficial effect of running the reaction under quasi‐homogeneous conditions. Under optimal reaction conditions (40 vol% of MeCN), the pseudo‐first order rate constant (*k*) of 4MBA oxidation was increased by a factor of 2.3 and 6.5 compared to the results for K,Na‐PHI and for a conventional CN photocatalyst in H_2_O, respectively. Furthermore, selectivities towards the reaction products 4MBAL (91 %) and H_2_O_2_ (76 %) were enhanced (Figure 5 a, Figure S18, Table S6). We point out that at lower conversion degrees (50 %) of 4MBA the selectivity is about 97 % and at even lower conversion levels it reaches up to 100 % (Figure S18, Table S6). It should also be noted that the catalytic performance of the solid heterogeneous photocatalysts, conventional CN and K‐PHI‐S, is practically not affected by the presence of the organic solvent in the reaction mixture (Figure S22).

Interestingly, the quasi‐homogeneous nature of the photocatalytic systems allows to reduce the scattering effects on the suspended particles compared to conventional heterogeneous photocatalysis, improving the reliability of quantum yield estimations for this system (see the Supporting Information). The K,Na‐PHI photocatalysts exhibited a quantum yield of about 10 % at 365 nm towards the 4MBAL formation, which is a rather respectable value as compared to common heterogeneous photocatalysts that typically show quantum yields of few per cent.^27^

A typical shortcoming of the practical implementation of homogenous or quasi‐homogeneous photocatalysts concerns difficult recovery for reuse. We find that our water soluble K,Na‐PHI photocatalyst can be easily recovered by increasing the ionic strength of the solution which causes coagulation and precipitation of K,Na‐PHI. This can be achieved by addition of a salt such as NaCl (Figure 1, Figure S10). The photocatalyst can then be centrifuged, separated from the liquid phase, and re‐dissolved. With this protocol, the photocatalyst can be reused in water as well as in H_2_O/MeCN(40 vol. %) medium for at least eight reaction cycles without any deterioration of its activity or selectivity (Figure 5 b, Figure S23).

Note that the superior photocatalytic performance of the water‐soluble K,Na‐PHI photocatalyst, compared to conventional solid PCN, is not limited to oxidation of 4MBA. Also, benzyl alcohol and lignocellulose‐derived feedstocks, such as ethanol, glycerol, and glucose,^28^ can act as effective hole scavengers. Using our water‐soluble PCN photocatalyst, the H_2_O_2_ production was enhanced, as compared to the conventional solid PCN, by the factors of 3.9, 3.2, 2.8, and 6.8 in reactions with benzyl alcohol, ethanol, glycerol, and glucose as reducing agents, respectively (Figures S24, S25).

**Figure 6:**
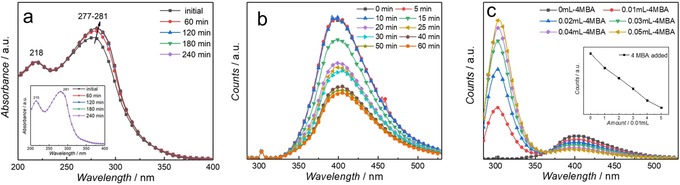
a) in situ UV/Vis spectra of the K,Na‐PHI in H_2_O recorded during illumination at 365 nm under aerobic and O_2_‐free (inset) conditions. b) in situ steady‐state emission spectra of K,Na‐PHI recorded upon fluorescence excitation at 275 nm during external illumination at 365 nm every 5 min in water before (0–10 min) and after applying O_2_ (10–60 min). c) in situ steady‐state emission spectra of K,Na‐PHI (*λ*_ex_=275 nm) dissolved in water with addition of 4MBA (5 mm) during 365 nm illumination. Inset: maximum intensity of the emission band at 402 nm (associated with K,Na‐PHI emission) upon addition of various amounts of 4MBA.

In situ absorption and emission spectroscopy was performed to gain further insight into the photocatalytic performance of the water‐soluble K,Na‐PHI photocatalyst. Figure 6 a shows stable absorption spectra of K,Na‐PHI over 4 h, when dissolved in degassed water. Under ambient atmosphere, a slight change of the absorption band at around 280 nm is apparent during the first hour of irradiation. Subsequently, the photoinduced changes in the colloidal solution level off, reaching a state in which the absorption spectrum changes no further. Similar changes are observed in in situ emission spectroscopy, in which the sample is continuously irradiated by an LED at 365 nm, while the fluorescence is intermittently (every 5 min) excited at 275 nm (Figure 6 b). In the absence of atmospheric oxygen (spectra recorded at 0, 5, and 10 min) the emission intensity is stable. Upon exposing the sample to air, the emission intensity drops in the course of the first hour, after which the changes level off. These data point to the colloidal nanoparticles solution being a dynamic system in which a stable stationary state develops over time when the system is irradiated while in contact with air. Figure 6 c shows a fluorescence titration of K,Na‐PHI in water upon addition of 4MBA. Upon addition of the substrate, the emission band associated with the alcohol itself (at 300 nm) increases, while the emission of the K,Na‐PHI decreases, indicating interaction of the photoexcited photocatalyst with the substrate. The fact that both dioxygen and 4MBA quench effectively the emission of the photoexcited K,Na‐PHI corroborates its high activity in simultaneous photooxidation of 4MBA and reduction of O_2_ to H_2_O_2_.

The high photocatalytic activity of the K,Na‐PHI photocatalyst along with its solubility in water represent a great promise for its use in light‐driven valorization of low‐value feedstock such as insoluble lignocellulosic substrates,^29^ where conventional solid‐state CN materials would be inefficient due to their low surface area hampering the accessibility of substrates to the active sites. In this context, it is noteworthy that catalysts of PCN family are not only capable of oxidation of benzylic C‐OH, but also of benzylic ‐CH_3_ oxidation reactions in model lignin compounds.^30^ Finally, we note that while the K,Na‐PHI is fully water‐soluble in a wide pH range (5–13), it can be converted into a gel simply by adding a strong acid, such as HCl. Therefore, we anticipate that the here presented water‐soluble carbon nitrides can be utilized not only in any applications requiring aqueous liquid processing of carbon nitride, but also as building blocks in fabrication of further novel photocatalytic architectures with tailored morphology and porosity using sol‐gel chemistry.

## Conclusion

A facile low‐temperature bottom‐up synthesis of water‐soluble heptazine‐based polymeric carbon nitride exhibiting high photocatalytic activity and selectivity is reported for the first time. Our structural investigations and theoretical calculations suggest that the water solubility and excellent stability of colloidal solutions of K,Na‐poly(heptazine imide), in contrast to conventional insoluble PCN materials, is conditioned by a decreased number of terminal NH_*x*_‐groups which are exchanged for cyamelurate moieties. These negatively charged surface functional groups interact preferentially with water, in particular with water molecules of the tight hydration shell of Na^+^ ions, which, in turn, leads to formation of an effective hydration layer on the surface of K,Na‐PHI nanoparticles and thus precludes the hydrogen‐bonding between the heptazine units that would otherwise cause coagulation and precipitation. The superior performance of these K,Na‐PHI nanoparticles compared to conventional solid PCN, is demonstrated in photocatalytic synthesis of H_2_O_2_, via reduction of oxygen accompanied by highly selective (up to 100 %) photoreforming of 4‐methoxybenzyl alcohol, benzyl alcohol, and biomass‐derived feedstocks (ethanol, glycerol, glucose). The dissolved photocatalyst can be easily separated from the reaction medium by increasing the ionic strength of the solution, and repeatedly used in consecutive reaction cycles without any change in particle size or any noticeable loss in activity or selectivity. This work thus establishes a new method of easily operable quasi‐homogeneous photocatalysis with carbon nitrides and paves the way for the use of water‐soluble carbon nitrides in applications in which liquid aqueous processing or operation is mandatory.

## Conflict of interest

The authors declare no conflict of interest.

## Supporting information

As a service to our authors and readers, this journal provides supporting information supplied by the authors. Such materials are peer reviewed and may be re‐organized for online delivery, but are not copy‐edited or typeset. Technical support issues arising from supporting information (other than missing files) should be addressed to the authors.

Supplementary
