# Supplementary material for: Water‐Soluble Polymeric Carbon Nitride Colloidal Nanoparticles for Highly Selective Quasi‐Homogeneous Photocatalysis
Source: Angew Chem Int Ed Engl. 2019 Nov 26;59(1):487–95. doi: 10.1002/anie.201913331 (PMC6973021; doi:10.1002/anie.201913331)
Supplement: Supplementary file 1 — Supplementary [file ANIE-59-487-s001.pdf]

Angewandte Chemie
Eine Zeitschrift der Gesellschaft Deutscher Chemiker

Supporting Information

Water-Soluble Polymeric Carbon Nitride Colloidal Nanoparticles for Highly Selective Quasi-Homogeneous Photocatalysis**

Igor Krivtsov, Dariusz Mitoraj, Christiane Adler, Marina Ilkaeva, Mariana Sardo, Luís Mafra, Christof Neumann, Andrey Turchanin, Chunyu Li, Benjamin Dietzek, Robert Leiter, Johannes Biskupek, Ute Kaiser, Changbin Im, Björn Kirchhoff, Timo Jacob, and Radim Beranek**

anie_201913331_sm_miscellaneous_information.pdf

Experimental Procedures

Materials and synthesis

Melamine (99 %) provided by Sigma Aldrich, KOH and NaOH (Merck, 99 %) were used for the syntheses of the photocatalysts. 4-methoxybenzyl alcohol (4MBA), 4-methoxybenzaldehyde (4MBAL) both with purity of 98% benzyl alcohol (99.8 %), benzaldehyde (99.5 %), glucose (99.5 %) were supplied by Sigma Aldrich while ethanol (99.8 %) and glycerol (99.5 %) were purchased from VWR and all these compounds were used for the photocatalytic tests. Titanium oxysulfate hydrate containing approx. 17 wt% of sulfuric acid ($TiOSO_4 \cdot H_2O \cdot H_2SO_4$) and aqueous solution of H_2O_2 were purchased from Sigma Aldrich and used for photometric determination of H_2O_2.Conventional heptazine-based polymeric carbon nitride (CN) was synthesized for comparison by thermal condensation of 30 g of melamine at 530 °C for 4h, after that the solid was powdered in agate mortar. The solid-state potassium poly(heptazine imide) (K-PHI-S) and water-soluble nanoparticles of potassium poly(heptazine imide) (K-PHI), sodium poly(heptazine imide) (Na-PHI), and mixed sodium-potassium poly(heptazine imide) (K,Na-PHI) were prepared by grinding 1.5 g of melamine with appropriate quantities of KOH, NaOH or a mixture of KOH-NaOH and heating it in a lid-covered crucible at a rate of 5 °C min^{-1} up to 330 °C, then it was kept for 2h at the reached temperature before being withdrawn from the furnace. The obtained solid was powdered and mixed with 100 mL of deionized water. In the case of the K-PHI-S sample, the suspended solid was centrifuged and washed twice with deionized H_2O before being put inside a cellulose membrane with a pore size of 3.5 kDa and dialyzed against H_2O for the purification followed by drying it for 24 h at 80 °C. For the preparation of the K-PHI, Na-PHI and K,Na-PHI samples, the same procedure was implemented with the difference that after the addition of 100 mL of H_2O the insoluble part was filtered out, firstly, by a paper filter and then by a 0.2 μm PTFE syringe filter. Eventually, the solution of the alkali metal poly(heptazine imide) nanoparticles was dialyzed and concentrated by evaporation at 60 °C to a concentration of 5 g L^{-1}, which was determined gravimetrically. In order to obtain solid sample of the water-soluble alkali metal poly(heptazine imide), the solution was gradually evaporated at 60 °C and then dried for 24 h at 80 °C. Table S1 summarizes the synthetic conditions applied for the preparation of the poly(heptazine imide) samples and the yields of the obtained materials.

Characterization

A Mettler–Toledo instrument (TGA/SDTA851^e) was used to investigate the formation of K,Na-PHI from the melamine mixture with KOH/NaOH. For this, a small portion of a precursor containing 1.5 g of melamine, 0.56 g (10 mmol) of KOH and 0.2 g of NaOH (5 mmol) was put in a platinum crucible and heated at a rate of 5 °C min^{-1} up to 330 °C in He atmosphere. Thermal decomposition of the dried alkali metal poly(heptazine imide) samples was carried out using the same equipment, but at a heating rate of 10 °C min^{-1} in O_2 flow (50 mL min^{-1}) from 25 °C to 1000 °C. XRD patterns were obtained using a Pananalytical X'pert PRO diffractometer equipped with a Pixel detector and operating at Cu Kα radiation. FTIR spectra were recorded by means of a Shimadzu IRTracer-100 spectrometer at a resolution of 4 cm^{-1} from the samples powdered and pressed into KBr pellets.

^{1}H, ^{13}C and ^{15}N magic angle spinning (MAS) solid-state NMR spectra of the K-PHI-S and the dried K,Na-PHI samples were acquired on a Bruker Avance III 400 spectrometer operating at B_0 field of 9.4 T with ^{1}H, ^{13}C, ^{15}N Larmor frequencies of 400.1/100.6/40.6 MHz, respectively. All experiments were performed on a double-resonance 4 mm Bruker MAS probe, except the ^{15}N cross-polarization MAS (CPMAS) measurements, in which a double-resonance 7 mm Bruker MAS probe was used. Samples were packed into 4 or 7 mm zirconia rotors. Spinning rates between 5 and 15 kHz were employed to record all spectra. ^{1}H, ^{13}C and ^{15}N chemical shifts are quoted in ppm from: TMS (0 ppm) and α-glycine (secondary reference, C=O at 176.03 ppm and NH_3^+ at 347.6 ppm on the nitromethane scale, respectively). Specifically, the ^{13}C MAS NMR spectra were acquired using a high-power decoupling single pulse sequence with the following conditions: 12 kHz spinning rate, a pulse width of 2.56

μs (45° flip angle) corresponding to 49 kHz, with ^1H SPINAL-64 decoupling at 80 kHz, recycle delay (RD) was 60 s. The ^1H-^{13}C CPMAS spectra were acquired at a MAS rate of 12 kHz using a ^1H 90° pulse length set to 3.2 μs (78 kHz); the CP step was performed with a contact time (CT) of 3500 μs using a 50−100% RAMP shape pulse on the ^1H (66 kHz) channel and using a 45 kHz square pulse on the ^{13}C channel; RD was 5 s. During the acquisition, a SPINAL-64 decoupling scheme was employed using a pulse length for the basic decoupling units of 6 μs at a rf field strength of 80 kHz. ^1H-^{15}N CPMAS spectra were typically acquired with the following parameters: MAS rate of 5 kHz, ^1H 90° pulse length of 4 μs employing a rf field strength of 63 kHz, the CP step was performed with a CT of 2000 μs using a 50−100% RAMP shape on the ^1H (57 kHz) channel and using a 34 kHz square shape pulse on the ^{15}N channel; decoupling with SPINAL-64 using a pulse length of 6.8 μs using a rf field strength of 63 kHz was applied, RD was 5 s. All spectra were acquired at ambient probe temperature. MagicPlot Pro software was used for the deconvolution of the chosen spectra. The R^2 values for fitting curves were always higher than 0.97.
In order to perform X-ray photoelectron spectroscopy (XPS), the powder samples K-PHI-S and K,Na-PHI were placed on top of a conductive double-sided carbon tape (Ted Pella Inc., USA) and fixed to the XPS sample holder. The measurements were performed using a UHV Multiprobe system (Scienta Omicron, Germany) with a monochromatic X-ray source (Al Kα) and an electron analyzer (Argus CU) with 0.6 eV energy resolution. Charge compensation during data acquisition was realized by an electron flood gun (NEK 150, Staib, Germany) at 6 eV and 50 μA. The background was subtracted and spectra were calibrated using the C1s peak (284.6 eV) before undergoing fitting using Voigt functions (30:70). A scanning electron microscope JEOL-6610LV equipped with an Oxford Instruments EDX detector was used to collect SEM images of the dry poly(heptazine imide) samples and determine their elemental composition. Particle size distribution of the water-soluble poly(heptazine imide) samples and their zeta-potentials were determined by means of dynamic light scattering (DSL) and electrophoretic mobility methods using a Zetasizer Nano ZS (Malvern Instruments Ltd., UK) operating at a detection angle of 173.5°. Diffusive-reflectance UV-vis spectra of solids were taken by a Shimadzu UV2600 UV-vis spectrophotometer. UV-vis spectra of the poly(heptazine imide) nanoparticles solutions were recorded using a Cary 60 (Agilent Technologies) spectrophotometer. Cyclic voltammogram of the K,Na-PHI material was recorded in the dark in acetonitrile solution of tetrabutylammonium hexafluorophosphate ($TBAFP_6$, 0.1 M) using a SP-300 BioLogic potentiostat (Caix, France) and a three-electrode cell composed of a platinum counter electrode and an Ag/AgCl (sat. KCl) reference electrode. The working electrode was prepared by drop casting of the K,Na-PHI nanoparticles solution on a glassy carbon substrate followed by gelation by adding conc. HCl. The potential values were recalculated and reported with respect to the normal hydrogen electrode (NHE). The images were recorded using the SALVE at 80kV acceleration voltage (where indicated) and the Philips CM20 at 200kV, both in HRTEM mode. The liquid sample was diluted (where indicated) in purified water (10 μl of sample in 1 mL of water), then drop cast onto copper TEM support grids with holey carbon film. The undiluted liquid samples were prepared by dipping of the TEM grid into the solution and drying. The solid samples were sonicated in ethanol and then drop cast onto TEM grids.

Photocatalytic measurements

Selective photocatalytic oxidation reactions of 4MBA to 4MBAL and benzyl alcohol to benzaldehyde were carried out for the K-PHI-S, K-PHI, Na-PHI, and K,Na-PHI samples, CN was used as a reference. For this purpose, the solid samples and the solutions of the poly(heptazine imide) nanoparticles were suspended in 20 mL of water or water-acetonitrile solution of 4MBA (or benzyl alcohol) (0.1 mmol). The pH of water solutions or water-acetonitrile mixture was in the range of 6.5–7.0 and did not undergo noticeable changes after irradiation. The reaction mixture was irradiated for 4 h by a UV LED emitting at 365 nm and producing a photon flux of 40 mW cm^{-2}. The control experiments were carried out in the absence of any photocatalyst under UV-irradiation and in the presence of the K,Na-PHI and CN samples in dark conditions. However, no noticeable reaction took place in such conditions, as the changes in the 4MBA or benzyl alcohol concentrations were below 1 %, and no H_2O_2 was detected after 4 h. The photocatalytic oxidation of 4MBA in the absence of air was performed under the optimal conditions (40 % *vol.*

acetonitrile in the reaction mixture), while purging Ar gas through the system. The samples of 0.1 mL were taken every 30 min and analyzed by an HPLC Shimadzu LC-10ADvp equipped with a UV detector SPD-20A and a reversed phase C18 Nucleosil column. Acetonitrile/water (30/70 vol.) was used as a mobile phase at a flow of 1.2 mL min^{-1} and a column temperature of 60 °C. 4MBA to 4MBAL were detected and quantified at a wavelength of 240 nm, while benzyl alcohol and benzaldehyde were measured at 220 nm. Considering a quasi-homogeneous nature of the irradiated 4MBA (or benzyl alcohol) solutions with the poly(heptazine imide) photocatalysts and the incompatibility of the used HPLC column with some of the organic solvent immiscible with water, which could possibly be used for the organic compounds extraction, the following probe preparation was implemented. The photocatalyst was separated from the reaction medium by means of a salting-out approach. In details, to 0.1 mL of the sample, 0.2 mL of 1M NaCl solution, and 0.8 mL of acetonitrile were added, the salting-out effect resulted in the two-phase system with the photocatalyst in the aqueous phase and 4MBA, 4MBAL (or benzyl alcohol and benzaldehyde) extracted to the organic phase, which was subsequently analysed. The respective volumes of the additives for the probe-preparation were adjusted for the case of acetonitrile/water solutions, as to maintain the H_2O to acetonitrile ratio in the probe constant. The calibration was carried out in the same way giving R^2 values higher than 0.999 for the substrate and the product. The recyclability test was made possible by the precipitation of the photocatalyst after each cycle by adding 0.25 g of NaCl to the reaction mixture in water solution and 0.125 to the reaction mixture in water/acetonitrile medium, following by its centrifugation and re-dispersion in a fresh solution of 4MBA. The selectivity (S) of the reaction was determined by the formula:

$$S_{4MBAL} = \frac{C(4MBAL)}{C_0(4MBA) - C(4MBA)} * 100\%,$$

where C_0(4MBA) is the initial concentration of the substrate, C(4MBA) is the substrate concentration at a given reaction time, and C(4MBAL) is the concentration of the reaction product at a given reaction time. Considering a *quasi*-homogeneous nature of the carried out photocatalytic reaction, the estimation of the quantum yield of the process is more reliable than for the case of heterogeneous systems. For this, the reaction mixture in water(60%)/acetonitrile(40%) medium containing 4MBA (5mM) and the K,Na-PHI photocatalyst having absorbance of 0.1 at 365 nm were put in a quartz cuvette and irradiated with a 365 nm emitting LED for 2 h. After the reaction the concentration of the produced 4MBAL was analysed using the above described HPLC method. The quantum yield (Q) of the process was calculated considering that two electrons are required to be withdrawn from the substrate (4MBA) to oxidize it to 4MBAL (1).

$$C_6H_3COH_3CH_2OH + 2h^+ \rightarrow C_6H_3COH_3CHO + 2H^+ \quad (1)$$
$$O_2 + 2H^+ + 2e^- \rightarrow H_2O_2 \quad (2)$$

The photon flux was determined by the formula:

$$\Phi = \frac{\Delta H}{qE},$$

where ΔH is the light power absorbed by the system (W m^{-2}), q is the charge of electron (C) and E is the energy of irradiation (eV).

The quantum yield was determined as:

$$Q = \frac{2\,\mathrm{n(4MBAL)}\,N_A}{\Phi t S},$$

where n(4MBAL) is the number of moles of 4MBAL produced, N_A is the Avogadro number, t is the irradiation time in s, and S is the irradiated area of the reactor (m^2)

Hydrogen peroxide concentration was estimated photometrically. Sample of 5 mL of the reaction solution was withdrawn after 4 h of irradiation, to that volume 1 mL of $TiOSO_4$ solution (Ti 1 wt%) in sulfuric acid was added.

The addition of the acidic solution caused the photocatalyst to coalesce, so it could be filtered out through the syringe PTFE 0.2 μm filter. The filtered solution of yellow-coloured titanium peroxocomplex was analysed photometrically using a Cary 60 (Agilent Technologies) spectrophotometer. The absorption at 420 nm was chosen to plot a calibration curve using standard H_2O_2 concentrations and to estimate the concentrations of photocatalytically produced H_2O_2. The selectivity towards the H_2O_2 formation was determined by the formula:

$$S_{H_2O_2} = \frac{C(H_2O_2)}{C_0(4MBA) - C(4MBA)} * 100\%.$$

The photocatalytic hydrogen peroxide production using model lignocellulose-derived substrates such as ethanol, glycerol or glucose was carried out in the same way as that of benzylic alcohols oxidation, however only H_2O_2 concentration was measured after 4 h of irradiation. The control experiments in the absence of photocatalysts or with CN and K,Na-PHI photocatalysts in the dark showed no H_2O_2 formation at such conditions. For the reactions with ethanol and glycerol 1 % wt. solutions of the substrates in water were prepared, while for the reaction with glucose 100 mg of it was dissolved in 20 mL of H_2O.

Theoretical calculations

Periodic Density Functional Theory (DFT) calculations were performed using the Vienna *Ab initio* Simulation Package (VASP) version 5.4.4[1–4] using the projector-augmented wave method[5] to represent the basis set. The exchange-correlation functional by Perdew, Becke, and Ernzerhof was used.[6] The wavefunction was optimized to an accuracy of 10^{-6} eV while geometries were relaxed until the forces reached below $5 \cdot 10^{-2}$ eV $Å^{-1}$. Gaussian finite-temperature smearing was employed with a smearing width of 0.01 eV. A plane wave energy cut-off of 400 eV and a 2x4x1 Monkhorst-Pack[10] ***k*** grid were found to give converged total energy results. 20 Å of vacuum separated slabs in ***z*** direction to avoid interaction between periodic images. For implicit solvation calculations, the GLSSA13 solvent model[7] implemented in the VASPsol extension was invoked.[8,9] We assumed typical room temperature bulk solvent dipole moments of 78.4, 36.6, and 32.6 for water, acetonitrile, and methanol, respectively.

Spectroscopic studies

In situ UV-Vis absorption and emission spectra were acquired in a 1cm quartz cell using a JASCO V-780 spectrophotometer and a fluorescence spectrometer (FLS980, Edinburgh), respectively. The data were recorded at room temperature upon irradiation of the sample with at 365 nm. External irradiation with the irradiance of 71 mW / mm^2 at the sample position was provided by a combined mounted LED (M365LP1, THORLABS). The K,Na-PHI sample was diluted in H_2O giving the optical density below 0.8 at 280 nm, and the freeze−pump−thaw method was used to remove oxygen for the measurements.

Results and Discussion

Synthesis

Table S1. Synthetic conditions for the poly(heptazine imide) samples preparation.

Sample name[a]	Melt composition	Sample state	Yield[c]	XRD phase composition[d]
		Potassium poly(heptazine imide)		
K-PHI-S1	KOH 5 mmol (0.28 g)	Mostly solid	27 %	PHI and melamine
K-PHI-S2	KOH 7.5 mmol (0.42 g)	Mostly solid	29 %	PHI and melamine
K-PHI-S3	KOH 10 mmol (0.56 g)	Solid and soluble PHI	23-26 %	PHI and melamine
K-PHI-S	KOH 10 mmol (0.56 g)	Dialyzed solid	24-25 %	PHI
K-PHI	KOH 12.5 mmol (0.70 g)	Dialyzed solution	10-11 %	PHI
K-PHI4	KOH 15 mmol (0.84 g)	Dialyzed solution	9 %	PHI
K-PHI5	KOH 20 mmol (1.12 g)	Dialyzed solution	3 %	PHI
		Sodium poly(heptazine imide)		
Na-PHI	NaOH 5 mmol (0.2 g)	Dialyzed solution	13 %	PHI
Na-PHI1	NaOH 7.5 mmol (0.3 g)	Dialyzed solution	4 %	PHI
Na-PHI2	NaOH 10 mmol (0.4 g)	Dialyzed solution	negligible	
		Potassium-Sodium poly(heptazine imide)		
K,Na-PHI1	KOH 5 mmol (0.28 g)<br>NaOH 5 mmol (0.2 g)	Dialyzed solution	6-9 %	PHI
K,Na-PHI2	KOH 7.5 mmol (0.42 g)<br>NaOH 7.5 mmol (0.3 g)	Dialyzed solution	12-18 %	PHI
K,Na-PHI3	KOH 10 mmol (0.56 g)<br>NaOH 10 mmol (0.4 g)	Dialyzed solution	9-11 %	PHI
K,Na-PHI4	KOH 10 mmol (0.56 g)<br>NaOH 7.5 mmol (0.3 g)	Dialyzed solution	7-9 %	PHI
K,Na-PHI	KOH 10 mmol (0.56 g)<br>NaOH 5 mmol (0.2 g)	Dialyzed solution	31-42 %	PHI

[a] The samples highlighted in grey are chosen for the investigation. [b] The materials were prepared by grinding 1.5 g of melamine with appropriate quantities of KOH, NaOH or a mixture of KOH-NaOH and heating it in a lid-covered crucible at a rate of 5 °C min^{-1} up to 330 °C, then it was kept for 2 h at the reached temperature before being withdrawn from the furnace. [c] The yield was quantified as the mass of the solid obtained after the synthesis (after washing or after dialysis) to the mass of melamine used for the synthesis and multiplied by 100 %. The yields depend on the presence of absorbed moisture in the hydroxide, hence may vary significantly [d] XRD patterns were collected from the dry solid samples, (PHI corresponds to alkali metal poly (heptazine imide) phase).

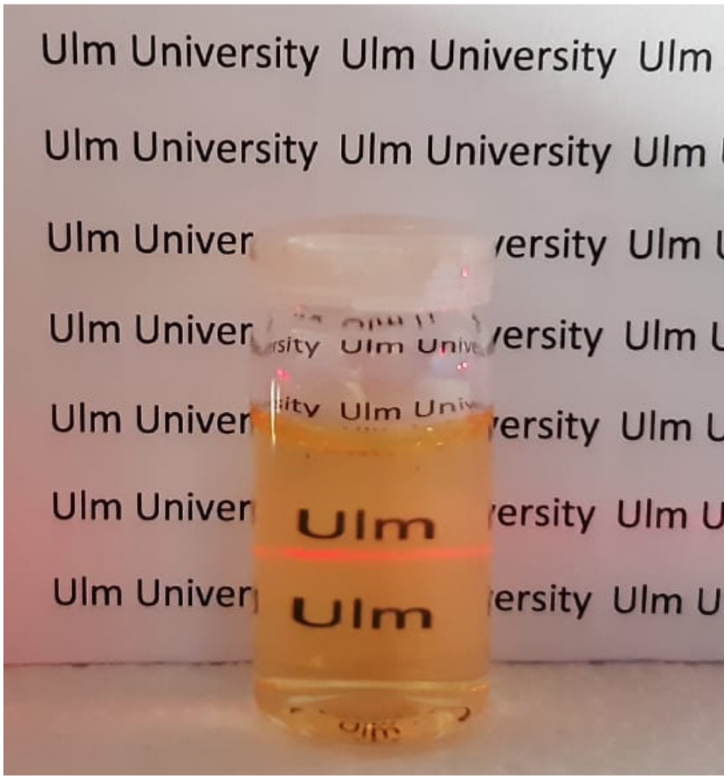

Figure S1. Tyndall effect observed in the solution of K,Na-PHI nanoparticles

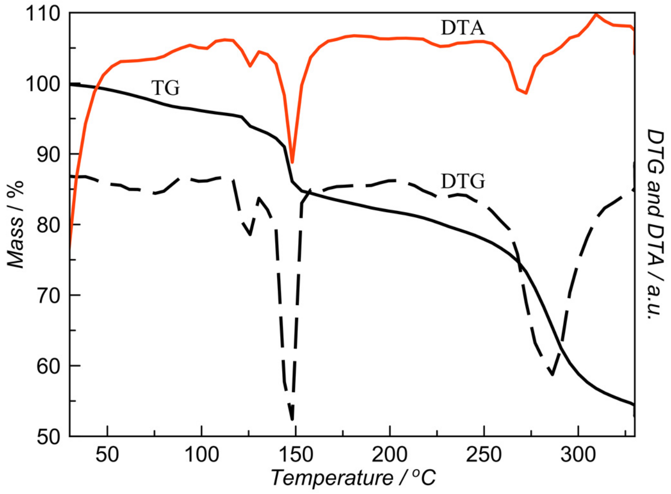

Figure S2. TG, DTG and DTA curves corresponding to the condensation process of melamine in KOH/NaOH mixture under He flow.

XRD analysis

The XRD patterns of the K-PHI-S sample series, obtained at different loadings of KOH in the melt, show that a part of melamine present in the precursor does not undergo the condensation to carbon nitride polymers, which is due to the fact that KOH is solid in this temperature range and it does not provide a liquid medium for melamine dissolution and condensation (Fig. S3 Left). Although the material recovered after the synthesis in KOH (K-PHI-S) was insoluble in water, its purification by washing and centrifugation or filtering was complicated owing to its high dispersibility in it. This is why the solid was dialysed, which allowed to achieve its higher purity (Fig. S3 Left).

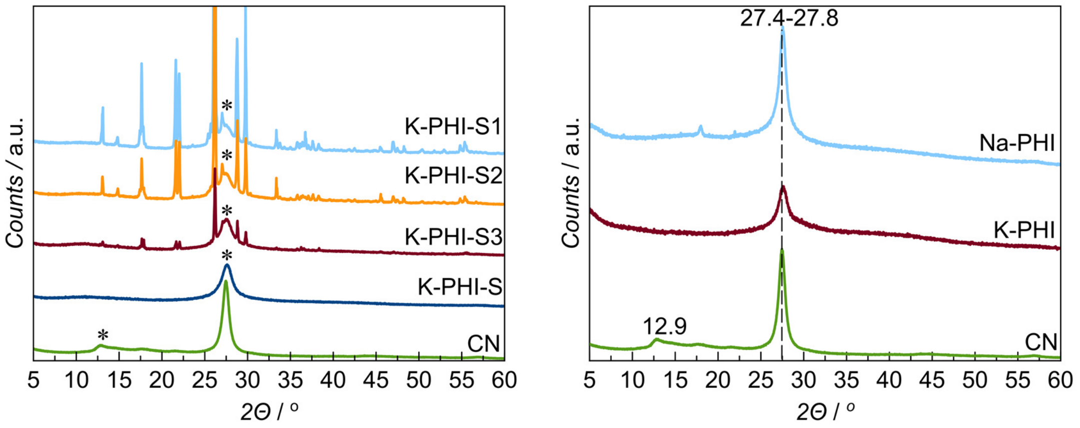

Figure S3. XRD patterns of the K-PHI-S samples (left) and the water soluble K-PHI and Na-PHI samples (right). Asterisk indicates the poly(heptazine imide) or PCN phases, while the other peaks correspond to non-condensed melamine.

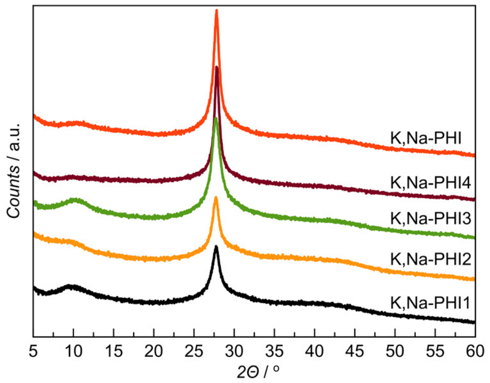

Figure S4. XRD patterns of the K,Na-PHI samples.

FTIR analysis

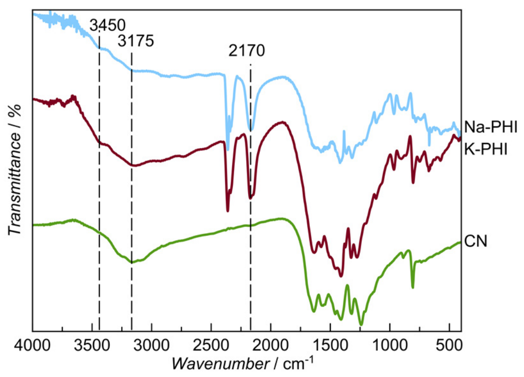

Figure S5. FTIR spectra of the K-PHI and Na-PHI samples in comparison with the CN sample.

TG-DTA analysis

The maximum of thermal decomposition rate of the K-PHI-S and K,Na-PHI samples is observed at about 690 °C (Fig. S6). A clear difference of thermal behaviour of the studied materials could be seen at high-temperature range. K-PHI-S loses 100 % of its initial mass by 800 °C, which it is not the case of K,Na-PHI retaining about 18 % of its initial mass at the same temperature (Fig. S6). The decomposition of the samples might result in the formation of potassium and sodium carbonates, hydroxides or oxides which are all not volatile compounds, however they melt at temperatures above 800 °C and then can be carried away by the gas flow (Fig. S6).

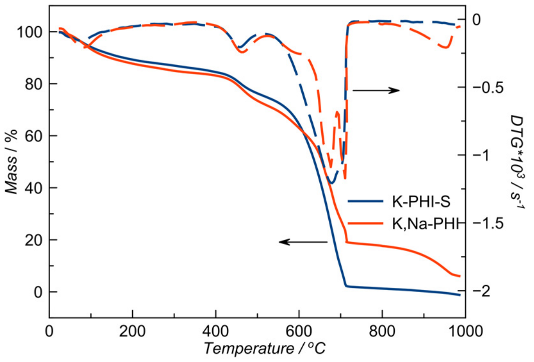

Figure S6. Thermoanalytical curves of the K-PHI-S and K,Na-PHI samples decomposition in O_2 (50 mL min^{-1}).

Elemental analyses

Table S2. Elemental composition of the prepared samples.

Sample name	Elemental composition / wt%[a]	C:N:H[a]	EDX Elemental composition / at% [b]	C:N ratio[b]
K-PHI-S	$C_{29.4}N_{50.5}H_{2.2}$	0.67:1.00:0.61	$C_{35.4}N_{56.1}O_{5.8}K_{2.7}$	0.63
K-PHI	-		$C_{35.6}N_{51.3}O_{8.6}K_{4.5}Ca_{0.1}$	0.69
Na-PHI	-		$C_{33.3}N_{54.2}O_{8.5}Na_{3.8}Ca_{0.2}$	0.61
K,Na-PHI	$C_{27.0}N_{44.4}H_{1.8}$	0.72:1.00:0.56	$C_{34.9}N_{47.8}O_{11.2}K_{2.5}Na_{3.5}Ca_{0.2}$	0.73

[a] On the basis of CHN elemental analysis. [b] Obtained by SEM-EDX method.

NMR study

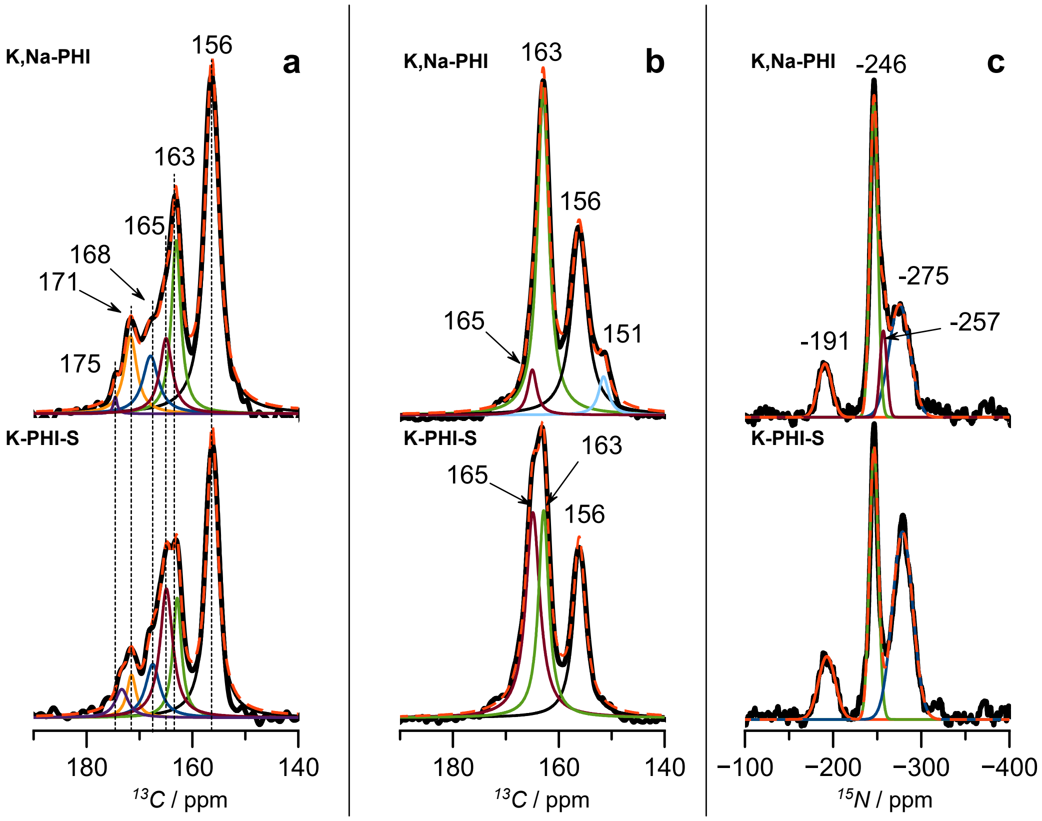

Figure S7. Peak fitting of the a) ^{13}C direct excitation MAS, b) ^{1}H-^{13}C CPMAS, and c) ^{1}H-^{15}N CPMAS solid-state NMR spectra of the K-PHI-S and K,Na-PHI samples. The experimental and fitted curves are represented in solid black and dashed red lines, respectively.

The ^{13}C direct excitation MAS spectra provide a quantitative estimation of the PCN species present in the studied samples (Table S3 and Fig. S7). The peak area of resonance **3** (associated to poly(heptazine) units) is used to estimate the relative amount of nitrile, C≡N (**1**), secondary amines, N=C-NH (**4**), primary amines, N=C-NH_2 (**5**), and oxygenated functional groups, N=C-O^- (**6**), between samples K,Na-PHI and K-PHI-S, by taking the ratios **1:3**, **4:3**, **5:3** and **6:3,** respectively (Table S3). The **6:3** ratio is similar in both materials (*cf.* 0.20 *vs.* 0.24) indicating that the relative amount of the C-O^- species is nearly identical for both. In contrast, the number of -NH_2 groups (**5:3** ratio) in the water-soluble K,Na-PHI decreases with respect to K-PHI-S (*cf.* 0.20 *vs.* 0.50, respectively), suggesting that the KOH/NaOH medium promotes a higher degree of condensation of the -NH_2 groups. Consequently, the formation of heptazine species (Fig. S8) is favoured, resulting in an increased number of N-C=N (**3**) carbons in K,Na-PHI as compared to K-PHI-S (Table S3, Fig. S7). The lower concentration of terminal -NH_2 moieties in K,Na-PHI will likely lead to an overall decrease of the number of hydrogen bonds in the polymer network, thus exerting a direct influence on aggregation and solubility.

Table S3. Estimation of relative concentrations of the PCN species from the deconvoluted ^{13}C MAS NMR spectra

Peak position	Assignment	Contribution of the peak area to the total area of the spectrum (FWHM)		Peak area ratio of the peaks with respect to the area of **3**	
		K-PHI-S	K,Na-PHI	K-PHI-S	K,Na-PHI
122 (**1**)	C≡N	0.13 (6.6)	0.15 (6.6)	0.34	0.35
156 (**3**)	N=C-N (C-N_3)	0.38 (2.6)	0.43 (3.0)	1	1
163 (**4**)	N=C-NH	0.12 (2.0)	0.15 (2.1)	0.32	0.35
165 (**5**)	N=C-NH_2	0.19 (2.9)	0.09 (2.9)	0.50	0.20
168 (**6**)	N=C-O^-	0.09 (3.3)	0.08 (3.5)	0.24	0.20
171-175 (**7**)	C-N^-	0.09 (2.1+2.9)	0.10 (2.9+1.0)	0.24	0.23

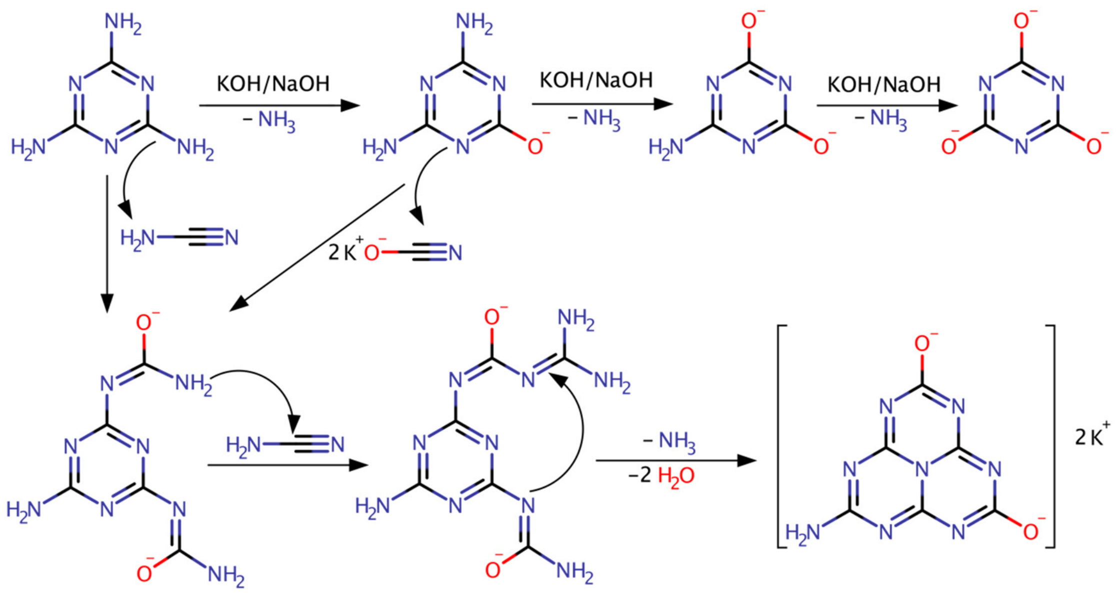

Figure S8. Proposed poly(heptazine imide) condensation pathway in KOH/NaOH melt.

XPS study

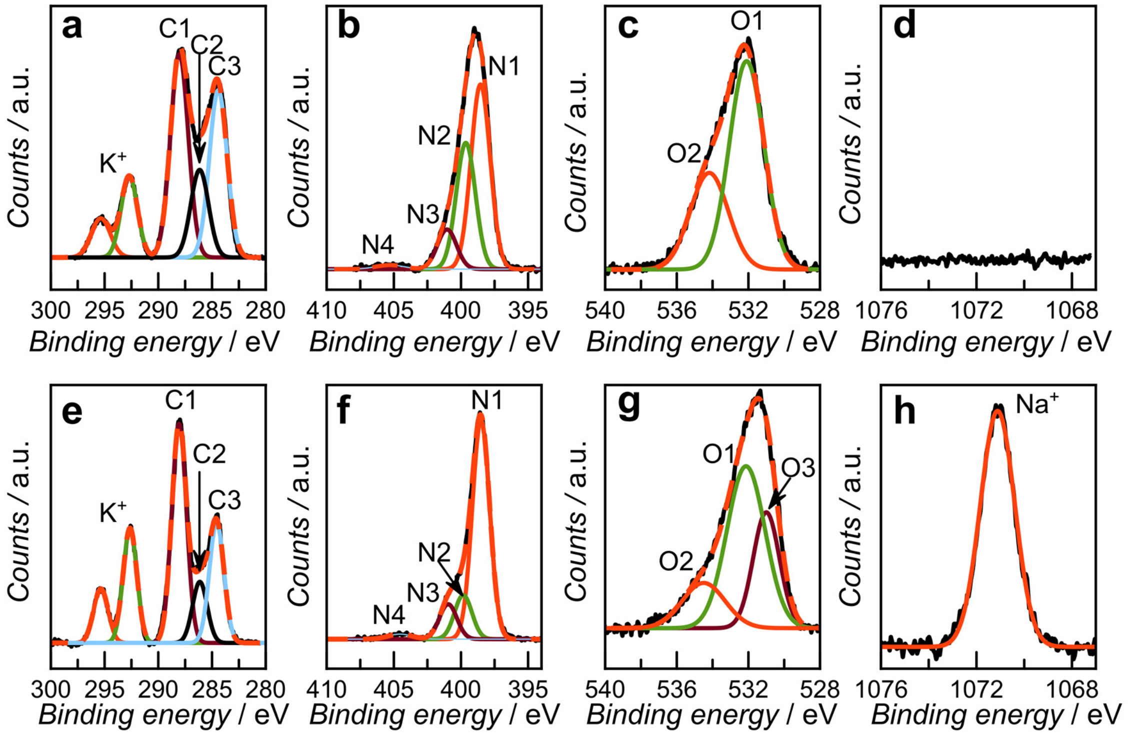

Figure S9. (a, b, c, d) and (e, f, g, h) XP C1s, N1s, O1s and Na1s spectra obtained for the K-PHI-S and K,Na-PHI samples, respectively.

Table S4. XPS data analysis.

Peak	Assignment	Peak position / eV	
		K-PHI-S	K,Na-PHI
C1	N-C=N, C=O	288.0	288.1
C2	C-OH, C-NH_2	286.2	286.1
C3	C-C from carbon tape and adventitious carbon	284.4	284.6
N1	C-N=C, C≡N	398.5	398.5
N2	N-C_3, triazine C-NH_x*	399.6	399.8
N3	C-NH_x	401.0	400.9
N4	N-N	405.3	405.4
O1	O=C-O	532.1	532.1
O2	H_2O	534.2	534.5
O3	HCO_3^-	-	531.0
Ratio between the areas corresponding to the N1:N2:N3 species			
		1:0.68:0.21	1:0.20:0.16

*Peak N2 cannot be assigned solely to the N-C_3 groups, since the ratio between the peak areas of N2:N1 in the K-PHI-S sample of 0.68:1 is far superior to the theoretically possible for the fully condensed polyheptazine structure (*ca.* 0.16:1). We attribute this peak also to the contribution from -NH_x bearing triazine fragments such as melamine.[11,12]

Theoretical study of K,Na-PHI

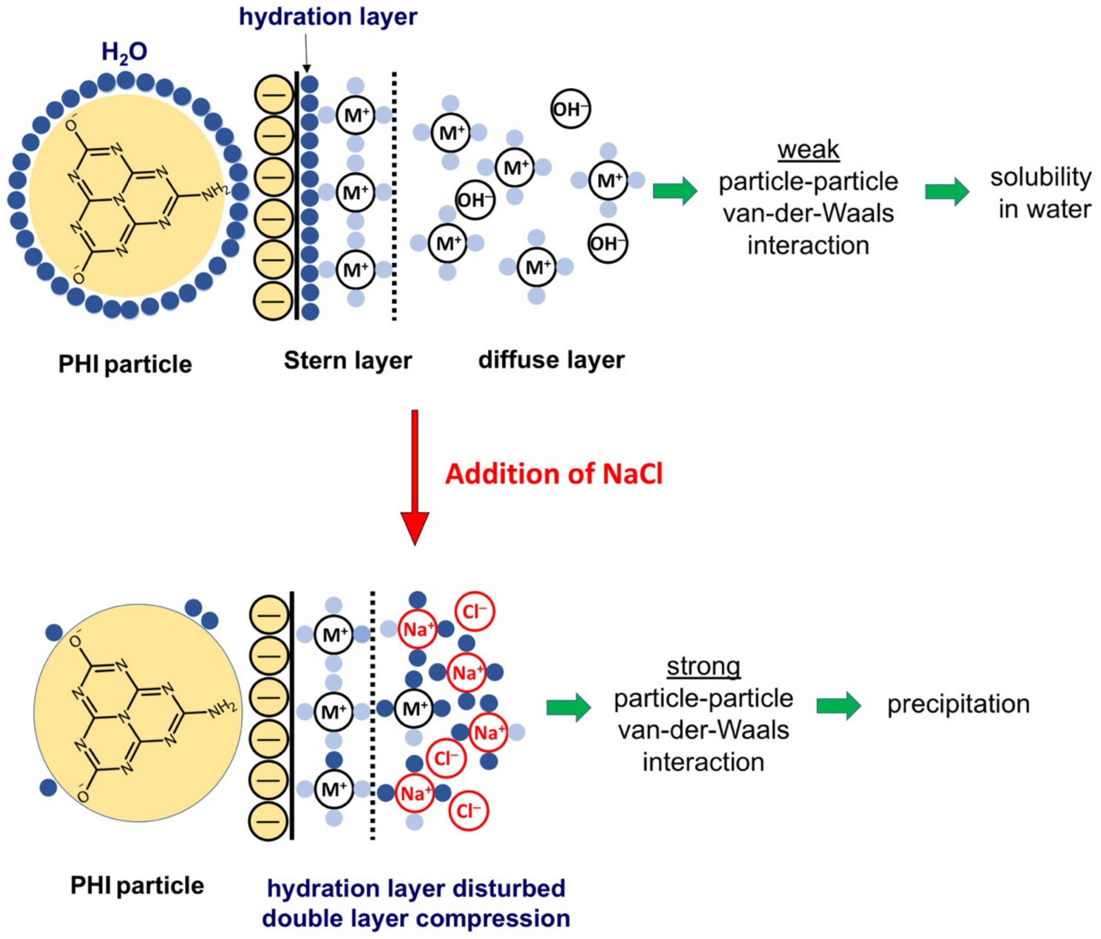

Figure S10. The salting out effect of NaCl addition on the double layer of the K,Na-PHI nanoparticles and their subsequent precipitation. At low ionic strength, the surface of PHI particles is covered with a hydration layer of water molecules, and the electric double layer extends over a relatively large distance, making the inter-particle van-der-Waals attractive forces weak. With increasing Na^+ concentration, due to the addition of NaCl, the hydration layer will gradually be stripped, disturbed as the water molecules hydrate preferentially the Na^+ cations; at the same time, the double layer is compressed due to the more effective screening by Na^+ ions. These effects result in stronger attractive particle-particle van-der-Waals interaction, leading to the coalescence of K,Na-PHI nanoparticles and their precipitation. The process is reversible: after centrifugation and removal of concentrated NaCl solution, the addition of water yields the PHI colloidal particles fully soluble again. M^+ stands for Na^+ or K^+.

The model system used for calculations is shown in Fig. S11 and is based on our experimental characterization; it exhibits a higher degree of condensation than typical carbon nitride model systems based on a periodic arrangement of melon strings but leaves enough boundaries to accommodate some functional groups to represent different N or O containing moieties.

Results of the solvation stabilisation study using the GLSSA13 implicit solvation model are summarized in Table S5. The stability of the model system in water, acetonitrile, and methanol is compared. Two main trends can be extracted from this data: (i) the water environment appears to stabilise the model system more strongly than the other two polar solvents, and (ii) stabilisation improves as a function of the number of oxygen-containing moieties.

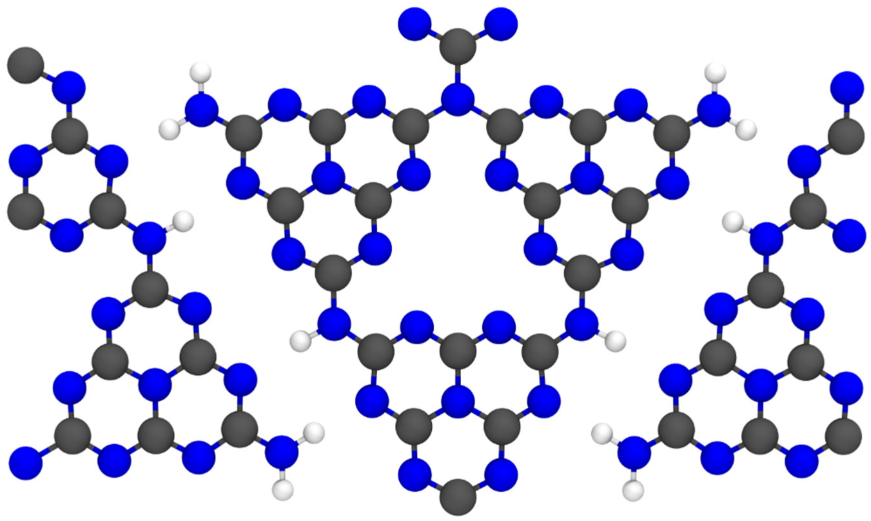

Figure S11. Illustration of one unit cell of the periodic computational carbon nitride model employed in the DFT study. The four -NH_2 groups are subsequently exchanged for -OH for the solvation study.

Table S5. Stabilisation of the carbon nitride model system in different implicit solvent models.

	Stabilization / eV vs. gas phase		
**# of NH_2 exchanged**	in water	in ACN	in MeOH
0	-1.46	-1.14	-1.10
1	-1.57	-1.24	-1.19
2	-1.66	-1.31	-1.26
3	-1.76	-1.40	-1.34
4	-1.86	-1.48	-1.42

DLS analysis

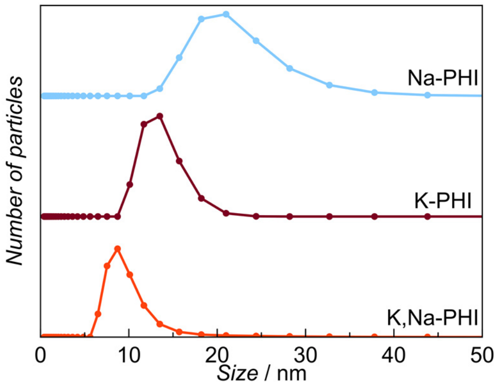

Figure S12. DLS particle size distribution of the poly(heptazine imide) samples.

SEM and TEM investigation

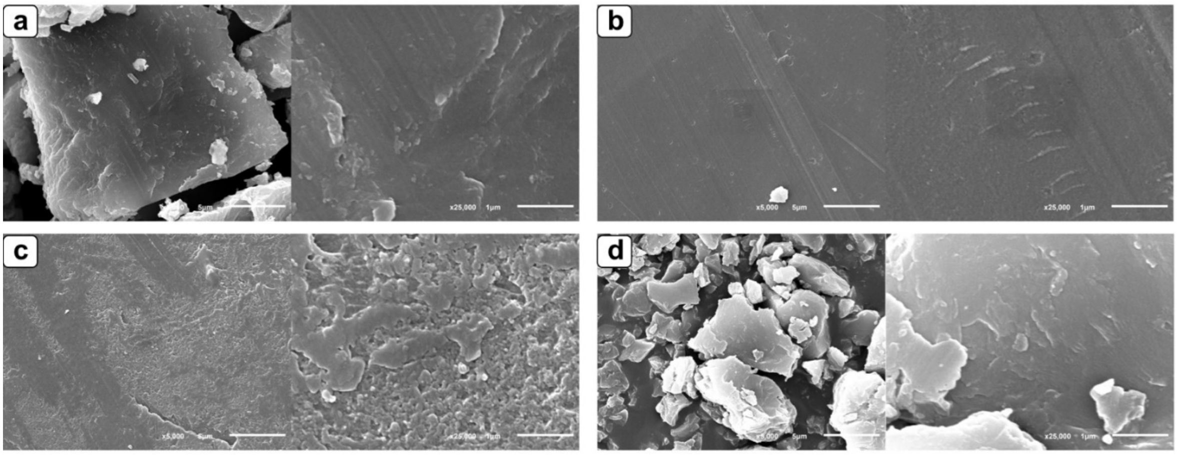

Figure S13. SEM images of the (a) K-PHI-S, (b) K-PHI, (c) Na-PHI and (d) K,Na-PHI samples.

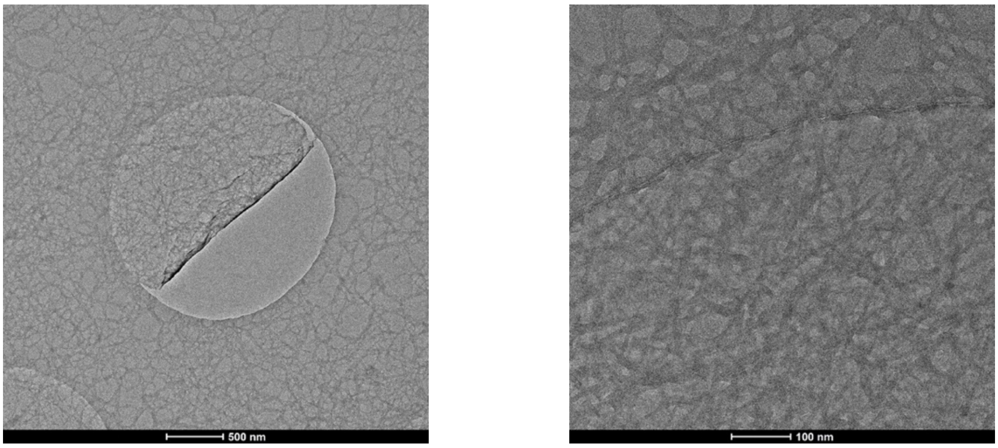

Figure S14. TEM images of the K,Na-PHI sample.

Photocatalytic studies

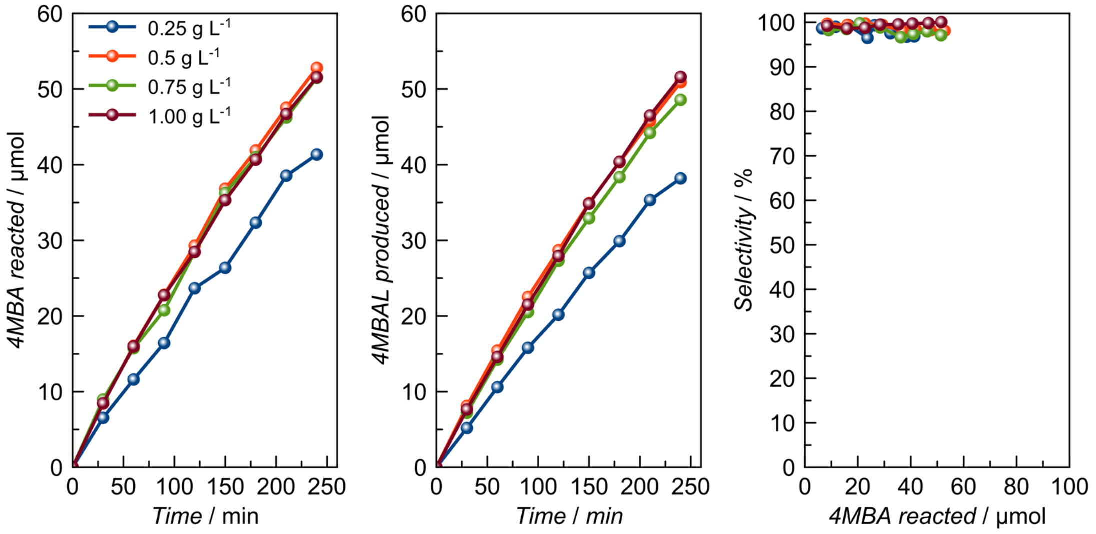

Figure S15. Photocatalytic 4MBA oxidation at different loadings of the K-PHI-S sample (20 mL H_2O, 4MBA 0.1 mmol, LED 365 nm).

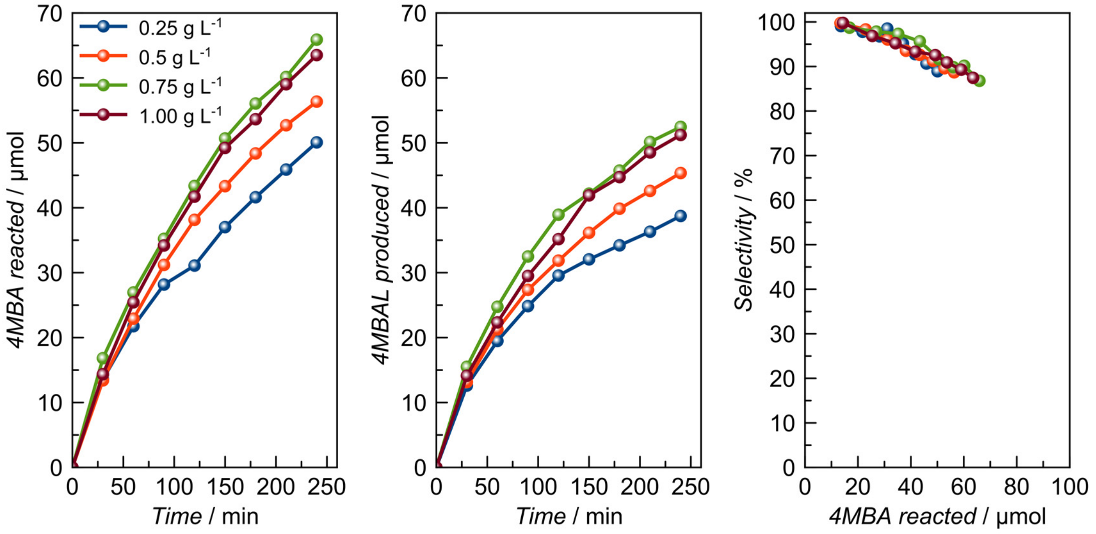

Figure S16. Photocatalytic 4MBA oxidation at different loadings of the K,Na-PHI sample (20 mL H_2O, 4MBA 0.1 mmol, LED 365 nm).

Table S6. Results of photocatalytic oxidation of 4MBA to 4MBAL with the concurrent H_2O_2 production

Sample	4MBA oxidation rate constant *10^3 / min^{-1}[a]	4MBA reacted in 4h / µmol	Selectivity to 4MBAL at 50% of 4MBA conversion / %	Selectivity to 4MBAL after 4 h / %	Selectivity to H_2O_2 after 4 h / %
CN H_2O	-1.4	28	-	≥ 99	97
K-PHI-S H_2O	-2.9	53	97	97	67
K,Na-PHI H_2O	-3.9	64	92	87	48
K,Na-PHI 10 % MeCN	-6.7	82	94	82	51
K,Na-PHI 20 % MeCN	-8.8	88	97	83	62
K,Na-PHI 40 % MeCN	-9.1	86	97	91	76
K,Na-PHI 50 % MeCN	-8.6	87	97	93	74
K,Na-PHI 70 % MeCN	-3.7	56	≥ 99	≥ 99	75
K,Na-PHI 85 % MeCN	-3.7	60	98	98	78

[a] The oxidation rate is quantified considering a pseudo-first order model with R^2 always ≥ 0.98

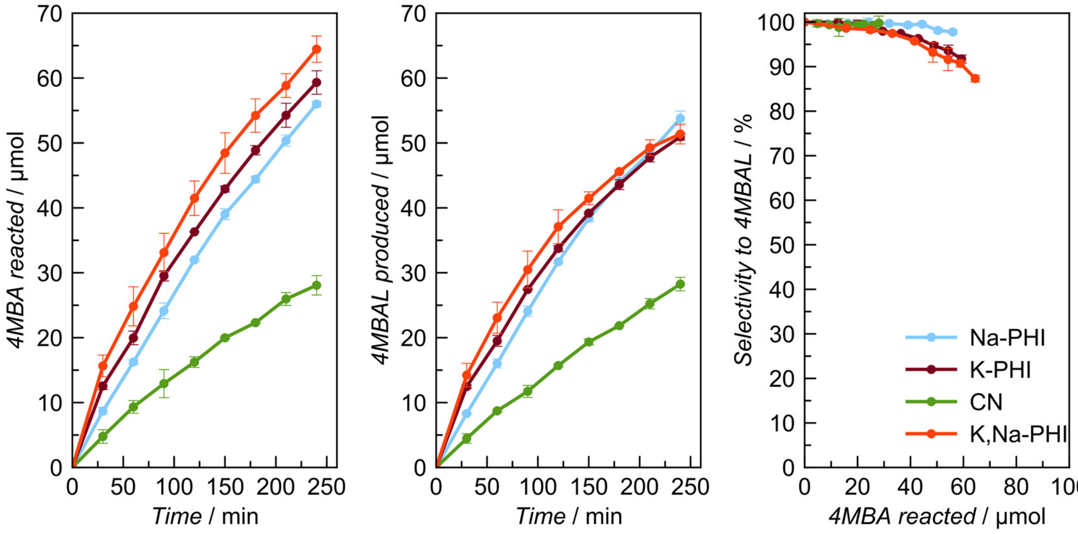

Figure S17. Photocatalytic 4MBA oxidation in the presence of K-PHI and Na-PHI samples (20 mL H_2O, 4MBA 0.1 mmol, LED 365 nm).

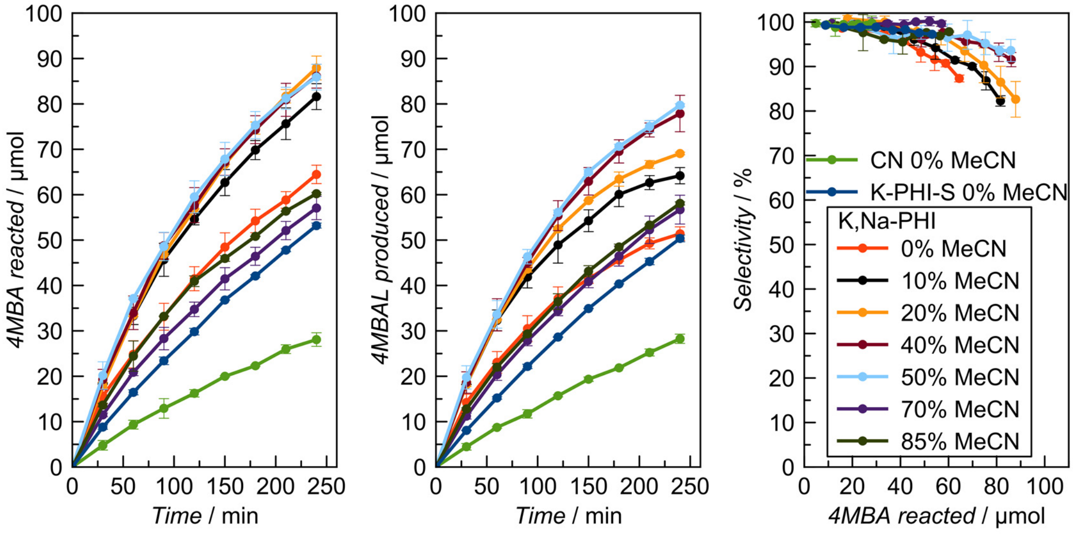

Figure S18. Photocatalytic oxidation of 4MBA in H_2O (20 mL, 4MBA 0.1 mmol, LED 365 nm) and in H_2O/MeCN mixture in the presence of K,Na-PHI (20 mL, X vol% of MeCN, 4MBA 0.1 mmol, LED 365 nm).

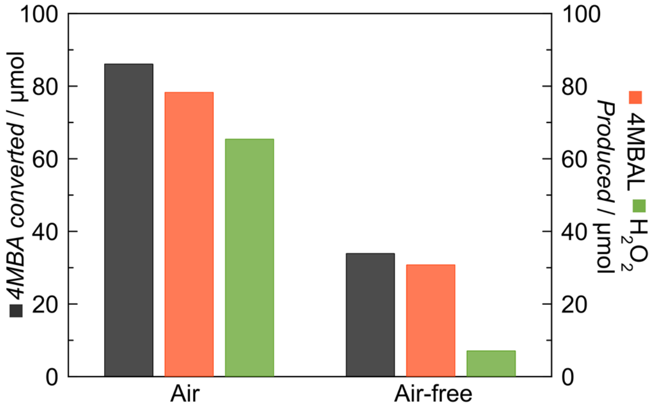

Figure S19. Conversion and selectivity of photocatalytic oxidation of 4MBA towards 4MBAL in air-free atmosphere (0.1 mmol 4MBA, 20 mL, H_2O/MeCN(40 vol.%), 365 nm, 4h) in presence of the K,Na-PHI photocatalyst.

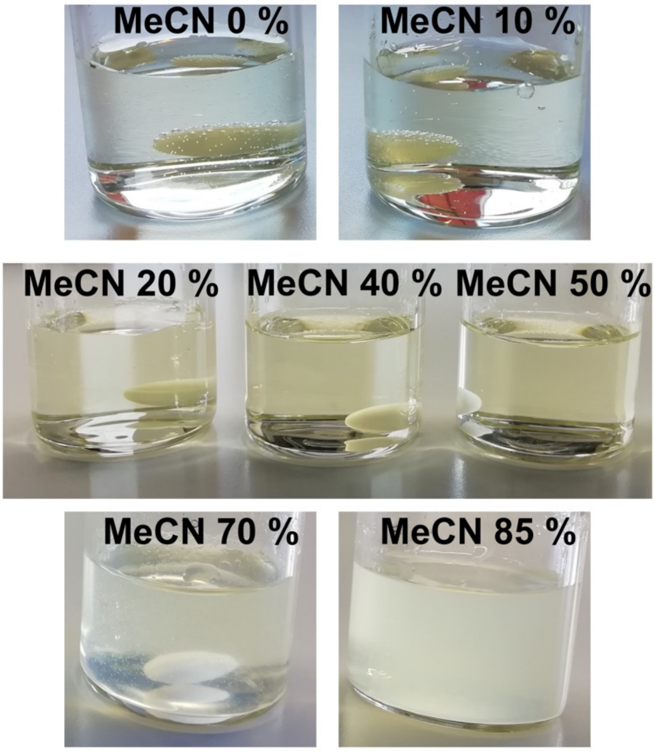

Figure S20. Photographs of the K,Na-PHI sample suspended in 4MBA solutions having varied MeCN vol. %.

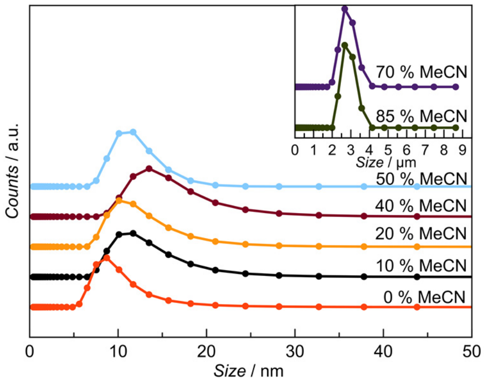

Figure S21. DLS particle size distribution of the K,Na-PHI sample suspended in 4MBA solutions having varied MeCN vol. %.

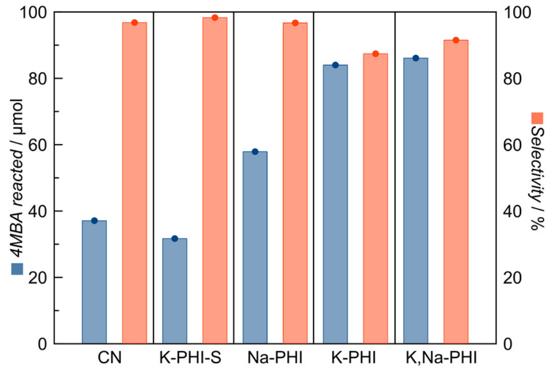

Figure S22. Conversion and selectivity of photocatalytic oxidation of 4MBA towards 4MBAL (0.1 mmol 4MBA, 20 mL, H_2O/MeCN(40 vol.%), 365 nm, 4 h) in presence of different photocatalysts.

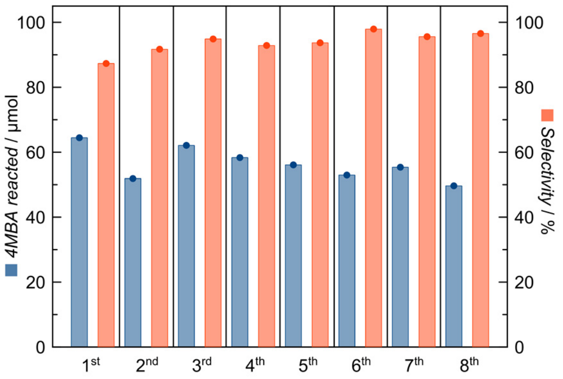

Figure S23. Recyclability test of the K,Na-PHI samples for 4MBA oxidation (20 mL, H_2O, 4MBA 0.1 mmol, LED 365 nm, 4 h of irradiation, 0.25 g of NaCl is added for the photocatalyst recovery).

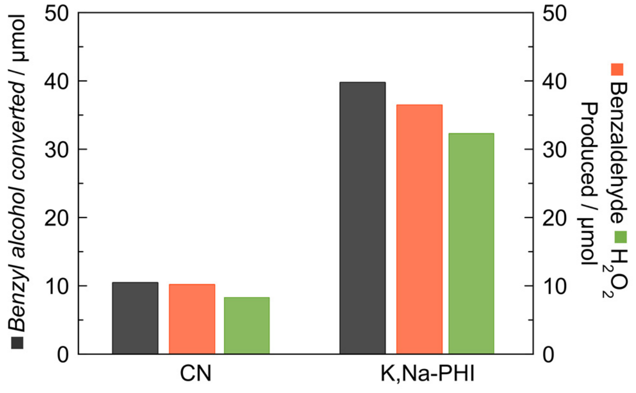

Figure S24. Conversion and selectivity of photocatalytic oxidation of benzyl alcohol towards benzaldehyde (0.1 mmol 4MBA, 20 mL of H_2O, 365 nm, 4 h) in presence of the CN and K,Na-PHI photocatalysts.

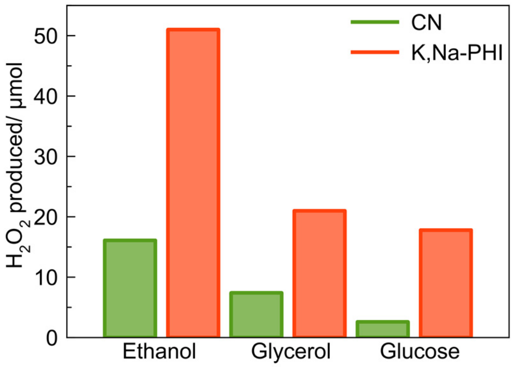

Figure S25. Photocatalytic H_2O_2 production using ethanol, glycerol and glucose as hole scavengers (ethanol and glycerol 1 wt. % solutions in 20 mL of H_2O, glucose 100 mg dissolved in 20 mL of water, 365 nm, 4h) in presence of the conventional CN and the water-soluble K,Na-PHI photocatalysts. The H_2O_2 production was enhanced by the factors of 3.2, 2.8 and 6.8 using ethanol, glycerol and glucose as reducing agents, respectively.

References

1. G. Kresse, J. Hafner, *Phys. Rev. B* **1993**, *47*, 558–561.
2. G. Kresse, J. Hafner, *Phys. Rev. B* **1994**, *49*, 14251–14269.
3. G. Kresse, J Furthmüller, *Comput. Mater. Sci.* **1996**, *6*, 15–50.
4. G. Kresse, J. Furthmüller, *Phys. Rev. B* **1996**, *54*, 11169–11186.
5. G. Kresse, D. Joubert, *Phys. Rev. B* **1999**, *59*, 1758–1775.
6. J. P. Perdew, K. Burke, M. Ernzerhof, *Phys. Rev. Lett.* **1996**, *77*, 3865–3868.
7. D. Gunceler, K. Letchworth-Weaver, R. Sundararaman, K. A. Schwarz, T. A. Arias, *Model. Simul. Mater. Sci. Eng.* **2013**, *21*, 074005.
8. K. Mathew, R. G. Hennig, *ArXiv160103346 Cond-Mat* (2016).
9. K. Mathew, R. Sundararaman, K. Letchworth-Weaver, T. A. Arias, R. G. Hennig, *J. Chem. Phys.* **2014**, *140*, 084106.
10. H. J. Monkhorst, *Phys. Rev. B* **1976**, *13*, 5188–5192.
11. A. P. Dementjev,et al. *Diamond Relat. Mater.* **2000**, *9*, 1904-1907
12. D. L. Zu, et al. *J. Mater. Sci.* **2007**, *43*, 689-695
